# Digital Quantitative Detection for Heterogeneous Protein and mRNA Expression Patterns in Circulating Tumor Cells

**DOI:** 10.1002/advs.202410120

**Published:** 2024-11-18

**Authors:** Hao Li, Jinze Li, Zhiqi Zhang, Qi Yang, Hong Du, Qiongzhu Dong, Zhen Guo, Jia Yao, Shuli Li, Dongshu Li, Nannan Pang, Chuanyu Li, Wei Zhang, Lianqun Zhou

**Affiliations:** ^1^ Suzhou Institute of Biomedical Engineering and Technology Chinese Academy of Science Suzhou 215163 China; ^2^ School of Biomedical Engineering (Suzhou) Division of Life Sciences and Medicine University of Science and Technology of China Hefei 230026 China; ^3^ The Second Affiliated Hospital of Soochow University Suzhou 215000 China; ^4^ Department of General Surgery Huashan Hospital & Cancer Metastasis Institute Fudan University Shanghai 200040 China

**Keywords:** Circulating tumor cells, d‐SCOUT, HCC management, mRNAs, multi‐real‐time digital PCR, proteins, simultaneous quantification

## Abstract

Hepatocellular carcinoma (HCC) circulating tumor cells (CTCs) exhibit significant phenotypic heterogeneity and diverse gene expression profiles due to epithelial‐mesenchymal transition (EMT). However, current detection methods lack the capacity for simultaneous quantification of multidimensional biomarkers, impeding a comprehensive understanding of tumor biology and dynamic changes. Here, the CTC Digital Simultaneous Cross‐dimensional Output and Unified Tracking (d‐SCOUT) technology is introduced, which enables simultaneous quantification and detailed interpretation of HCC transcriptional and phenotypic biomarkers. Based on self‐developed multi‐real‐time digital PCR (MRT‐dPCR) and algorithms, d‐SCOUT allows for the unified quantification of Asialoglycoprotein Receptor (ASGPR), Glypican‐3 (GPC‐3), and Epithelial Cell Adhesion Molecule (EpCAM) proteins, as well as Programmed Death Ligand 1 (PD‐L1), GPC‐3, and EpCAM mRNA in HCC CTCs, with good sensitivity (LOD of 3.2 CTCs per mL of blood) and reproducibility (mean %CV = 1.80–6.05%). In a study of 99 clinical samples, molecular signatures derived from HCC CTCs demonstrated strong diagnostic potential (AUC = 0.950, sensitivity = 90.6%, specificity = 87.5%). Importantly, by integrating machine learning, d‐SCOUT allows clustering of CTC characteristics at the mRNA and protein levels, mapping normalized heterogeneous 2D molecular profiles to assess HCC metastatic risk. Dynamic digital tracking of eight HCC patients undergoing different treatments visually illustrated the therapeutic effects, validating this technology's capability to quantify the treatment efficacy. CTC d‐SCOUT enhances understanding of tumor biology and HCC management.

## Graphical abstract

This study introduces the d‐SCOUT technology, which enables simultaneous quantification of mRNA and protein biomarkers in HCC circulating tumor cells. By integrating multi‐real‐time digital PCR and machine learning, d‐SCOUT provides detailed heterogeneous 2D molecular profiling for improved HCC diagnosis and treatment monitoring.

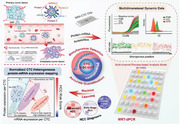

## 1 Introduction

Hepatocellular carcinoma (HCC), the most common form of liver cancer, poses a significant burden on the global healthcare system, with high mortality attributed mainly to late‐stage diagnosis and limited treatment efficacy.^[^ ^1^, ^2^ ^]^ Its high metastatic potential further complicates treatment.^[^ ^3^, ^4^ ^]^ Despite advancements in diagnostic imaging and molecular characterization, routine clinical practices such as periodic liver ultrasound with or without alpha‐fetoprotein (AFP) often lack sensitivity and fail to provide comprehensive insights into tumor biology.^[^ ^5^, ^6^ ^]^ Traditional approaches fall short in capturing the full complexity of tumor heterogeneity and dynamics.^[^ ^7^, ^8^ ^]^ Therefore, there is an urgent need for a high‐sensitivity method capable of offering detailed molecular insights, improving our understanding of tumor progression and treatment response.

In recent years, various liquid biopsy techniques have demonstrated immense potential for identifying cancer biomarkers, particularly circulating tumor cells (CTCs), circulating tumor DNA (ctDNA), and extracellular vesicles (EVs).^[^ ^9^, ^10^, ^11^, ^12^, ^13^, ^14^ ^]^ Among these, CTCs, shed from the primary tumor into the bloodstream, provide multidimensional dynamic information encompassing critical DNA, mRNA, and protein data from the tumor.^[^ ^15^, ^16^, ^17^, ^18^ ^]^ During metastasis, CTCs undergo significant phenotypic and gene expression changes due to epithelial‐mesenchymal transition (EMT) and mesenchymal‐epithelial transition (MET), manifesting in three typical protein expression patterns: overexpression, underexpression, and nonexpression.^[^ ^4^, ^19^ ^]^ These changes, coupled with patient variability, lead to substantial heterogeneity in CTC populations.^[^ ^20^ ^]^ Traditional methods,^[^ ^21^, ^22^, ^23^, ^24^ ^]^ which target specific protein markers, often miss underexpressing or nonexpressing tumor cells, particularly in highly metastatic tumors where EpCAM is downregulated during EMT, resulting in false negatives.^[^ ^3^, ^25^ ^]^ Single‐dimensional approaches fail to capture the full complexity of tumor heterogeneity, whereas multidimensional, multitarget detection offers a more comprehensive view of tumor progression and metastasis risk. Therefore, comprehensive multidimensional biomarker profiling of CTCs represents a highly anticipated strategy for future liquid biopsy applications.

Despite advances in HCC CTC identification, challenges persist in phenotype and molecular‐level detection. Current methods for protein detection, such as immunofluorescence and immunoblotting,^[^ ^22^, ^26^ ^]^ are limited in sensitivity and target range, providing only a broad assessment of CTC protein expression. Sequencing technologies for detecting CTC DNA mutations,^[^ ^27^ ^]^ copy number variations,^[^ ^28^ ^]^ and mRNA,^[^ ^29^, ^30^, ^31^ ^]^ though offering high throughput and resolution, lack phenotypic data and have limited correlation with clinical outcomes. Additionally, owing to their complexity and cost, they are not suitable for dynamic therapeutic monitoring. Consequently, current approaches do not provide unified, multidimensional molecular information, limiting their use in tumor staging and treatment evaluation. Although some novel platforms, such as folate‐mediated CTC quantitative PCR systems,^[^ ^32^, ^33^, ^34^ ^]^ HCC‐specific mRNA reverse‐transcription droplet digital PCR (ddPCR) systems,^[^ ^1^, ^8^, ^35^ ^]^ and single EV surface protein droplet digital immuno‐PCR (ddiPCR) systems,^[^ ^36^, ^37^ ^]^ have emerged, methods or platforms capable of simultaneously quantifying multiple phenotypic and genotypic or transcriptional markers in cancer samples are scarce. Key challenges include the absence of a unified platform for capturing intact CTC protein and mRNA and the lack of standardized criteria for their concurrent quantitative analysis.

Real‐time digital PCR (RT‐dPCR) is an emerging technique that enables real‐time monitoring of fluorescence signals for more accurate absolute quantification at the single‐molecule level, crucial for detecting low‐abundance protein or mRNA markers in disease diagnosis.^[^ ^38^, ^39^, ^40^ ^]^ Recently, Tay et al. developed a sensitive method for quantifying proteins and mRNA in single cells via dPCR, showing the potential for multidimensional biomarker detection.^[^ ^41^, ^42^ ^]^ However, their approach is not suitable for simultaneous multi‐protein and multi‐mRNA analyses to detect rare CTCs in complex blood samples. To address this challenge, our group has been exploring strategies for efficient CTC enrichment and simultaneous detection of protein and nucleic acid markers in HCC.^[^ ^43^, ^44^, ^45^ ^]^ Alongside advancements in RT‐dPCR technology,^[^ ^46^, ^47^ ^]^ we envision a novel approach for multiquantitative assessment of CTC proteins and mRNAs in HCC, providing accurate molecular insights for personalized diagnosis, monitoring, and treatment assessment.

In this study, we developed an HCC CTCs Digital Simultaneous Cross‐dimensional Output and Unified Tracking (d‐SCOUT) technology, based on multi‐channel radial cross‐flow CTC Chip (MRX‐CTC chip) and multi‐real‐time digital PCR (MRT‐dPCR). This system efficiently enriched CTCs while preserving proteins and mRNAs, allowing simultaneous quantitative analysis of CTC transcriptotypes and phenotypes. Using immuno‐PCR principles, we converted CTC protein detection into nucleic acid detection via molecular tags, enabling enhanced sensitivity and concurrent protein and mRNA analysis on the same platform. To streamline the process, we integrated a windmill‐like filter^[^ ^44^ ^]^ design into the MRX‐CTC chip, which enables continuous CTC enrichment, labeling, and biomarker collection without sample transfer, minimizing nonspecific adsorption and cross‐contamination. To assemble our diagnostic arsenal for HCC, we strategically selected for three protein markers – EpCAM, GPC‐3, and ASGPR – driven by clinical imperatives for enhanced diagnostic potential. Additionally, we integrated three mRNA markers, facilitating the study of EpCAM and GPC3, which correlate with their respective proteins, alongside PD‐L1, serving as a therapeutic guide. We further optimized the MRT‐dPCR workflow to provide precise, multidimensional quantitative outcomes. Validation with HCC cell lines (HuH‐7 and Hep3B) confirmed the platform's reliability. We also established a comprehensive digital scoring system for HCC CTC biomarkers, validated across clinical cohorts including HCC patients, healthy volunteers, post‐treatment HCC patients, and patients with other cancers. Additionally, machine learning was employed to map heterogeneous protein and mRNA expression patterns in CTCs, enabling insights into metastasis and therapeutic response. Our CTC d‐SCOUT technology functions like a skilled reconnaissance scout, offering in‐depth, multidimensional molecular data that helps clinicians develop more precise, personalized treatment plans, improving diagnostic accuracy and advancing personalized HCC management.

## 2 Results

### 2.1 The Workflow of HCC CTC d‐SCOUT for Phenotypic and Transcriptional Analysis

**Figure 1:**
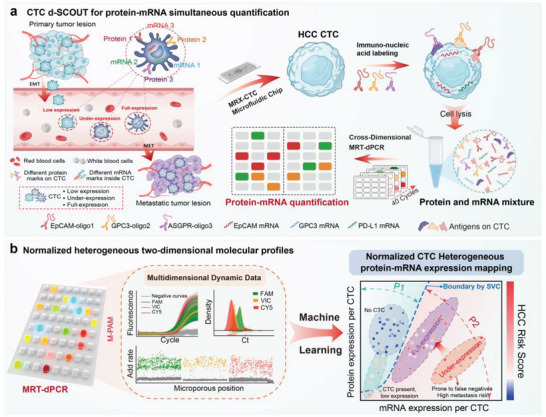
Simultaneous acquisition and quantification of HCC CTC transcriptotypic and phenotypic biomarkers for personalized HCC management. a) Workflow overview of CTC d‐SCOUT technology for simultaneous quantification of HCC CTC transcriptotypic and phenotypic. Venous blood is pretreated in the MRX‐CTC chip to isolate mRNA and protein biomarkers. Leveraging the immuno‐PCR principle, molecular tags are employed to allow the detection of HCC CTC protein markers via nucleic acid detection. This process provides high retention of mRNA markers and target protein–oligos in the MRX‐CTC chip, which is then integrated with our cross‐dimensional MRT‐dPCR to achieve accurate and simultaneous quantitative analysis of HCC CTC transcriptotypes and phenotypes. b) Normalized heterogeneous two‐dimensional molecular profiles. Downstream analysis is performed via MRT‐dPCR, which is integrated with the M‐PAM algorithm to obtain dynamic multi‐dimensional biomarker data. These data include amplification curves of all microwells, Ct values distributions, and fluorescence growth rates. The information can be further utilized in machine learning‐based classification to normalize and analyze the heterogeneity of CTC protein‐mRNA expression profiles, aiding in the diagnosis, progression monitoring, and therapeutic responses evaluation in HCC.

The workflow of CTC d‐SCOUT technology for simultaneous quantitative analysis of protein and mRNA in HCC is briefly outlined in **Figure** **1**. Leveraging immuno‐PCR, with unique molecular tags specifically bound to protein markers of HCC CTCs (EpCAM‐oligo1, GPC3‐oligo2, and ASGPR‐oligo3), well‐preserved mRNA markers and quantified protein oligos were obtained from the MRX‐CTC chip (Figure S1, Supporting Information), which was ultimately integrated with our cross‐dimensional MRT‐dPCR to achieve simultaneous and accurate absolute quantification of transcriptotypes and phenotypes (Figure 1a). The three sets of oligos and corresponding primer and probe sequences, along with the primer and probe sequences for three sets of mRNA targets designed to quantify multiple CTC proteins and mRNAs, are detailed in Table S1 (Supporting Information). All primers demonstrated amplification efficiencies greater than 90% (Figure S2, Supporting Information). Dynamic multidimensional biomarker data generated by MRT‐dPCR, with the assistance of machine learning, enable further digital two‐dimensional molecular mapping analysis, which supports the diagnosis, monitoring, and evaluation of therapeutic responses in HCC (Figure 1b). The newly developed d‐SCOUT technology enables the concurrent quantification of three surface proteins and three mRNAs in rare CTCs within HCC blood samples, providing a more comprehensive understanding of the tumor profile for the personalized management of HCC.

### 2.2 Characterization of Liver Cancer Cell Line Proteins and mRNAs

**Figure 2:**
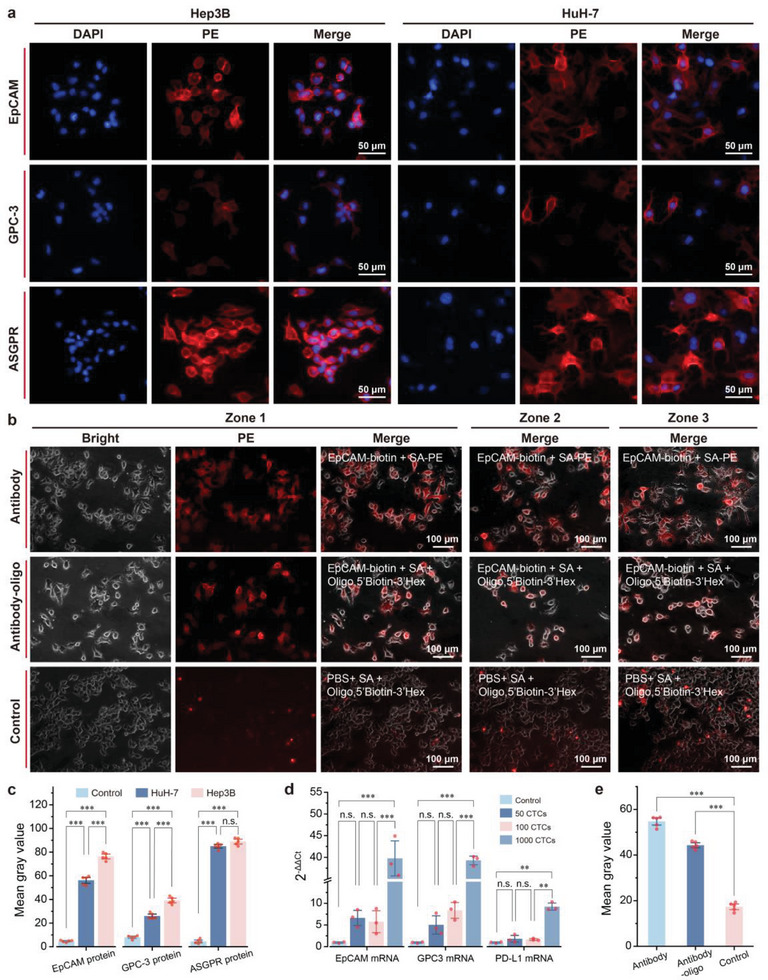
Characterization of protein and mRNA markers in HCC cell lines and feasibility evaluation of protein‒mRNA codetection. a) Representative fluorescence images of EpCAM, GPC‐3, and ASGPR protein expression in Hep3B and HuH‐7 cells. b) Visualization of the specific binding of antibody‒oligo conjugates to HCC cell lines. Fluorescence images of Hep3B cell lines were captured after treatment with PE‐tagged EpCAM, the Hex‐tagged EpCAM‐oligo conjugate, or the Hex‐tagged oligo to assess the effectiveness of the self‐synthesized antibody‒oligo conjugate. c) Comparison of grayscale values (fluorescence intensity) of different protein markers in HCC cell lines on the basis of their fluorescence images. The control groups were treated with PE‐labelled streptavidin. The data are presented as the means ± SDs of 5 independent assays. d) Relative quantitative comparison of different mRNA markers at various concentrations in HCC CTCs via real‐time PCR. The data are presented as the means ± SDs of 3 independent assays. e) Comparison of grayscale values of fluorescence images of HCC cell lines after antibody‐PE, Hex‐labelled antibody‐oligo, and Hex‐labelled oligo treatment. The data are presented as the means ± SDs of 5 independent assays. Significant differences between groups were assessed via one‐way ANOVA.

To establish a reference framework for the subsequent application of our novel method, we focused on two liver cancer cell lines, HuH7 and Hep3B, and performed a comprehensive evaluation of their protein and mRNA expression levels. **Figure** **2**a shows the results of qualitative immunofluorescence experiments targeting multiple antigens, revealing the presence of EpCAM, ASGPR, and GPC3 in both cell lines. Notably, the expression levels of ASGPR and EpCAM were consistently greater than those of GPC3 across both cell lines. To precisely quantify protein expression in HuH7 and Hep3B cells, we calculated and analyzed grayscale values representing fluorescence signal intensity from fluorescence images obtained from various cellular regions. As depicted in Figure 2c, the fluorescence signal intensities of EpCAM, ASGPR, and GPC3 in both HuH7 and Hep3B cells significantly exceeded those of the control group (***,*p* < 0.001). Furthermore, the fluorescence signal intensity of EpCAM and GPC3 in Hep3B cells surpassed that in HuH7 cells (***,*p* < 0.001), whereas the ASGPR fluorescence intensity did not significantly differ between Hep3B and HuH7 cells. For the evaluation of mRNA expression levels in HCC CTCs, qRT‒PCR analysis was conducted on samples containing 0, 50, 100, and 1000 CTCs, with relative quantification performed via the 2^−ΔΔCt^ method.^[^ ^7^, ^48^ ^]^ Figure 2d summarizes the relative expression levels of EpCAM, GPC3, and PD‐L1 mRNAs across different CTC counts. Notably, the expression levels of EpCAM, GPC3, and PD‐L1 mRNAs were significantly greater in samples from 1000 CTCs than in those from fewer than 100 CTCs. Additionally, PD‐L1 mRNA exhibited a lower relative expression level than the other two mRNAs did.

### 2.3 Visualization of Antibody‒Oligo Conjugates

To enable visual tracking of the antibody‒oligo conjugates used for CTC‐specific recognition, the antibody‒oligo conjugates were labeled with Hex dye. As shown in Figure 2b,e, specific recognition by antibodies and antibody‒oligo conjugates resulted in a significantly higher (***,*p* < 0.001) average grayscale value than in the control group. Additionally, the average grayscale value of antibody‐oligo labeling was only slightly lower (by 10.4) than that of standard antibody staining (Figure 2e). This finding demonstrated that coupling via streptavidin and biotin layer‐by‐layer assembly effectively labels cells with oligos for quantitative protein analysis.

### 2.4 Design and Performance Evaluation of the Integrated MRX‐CTC Chip

**Figure 3:**
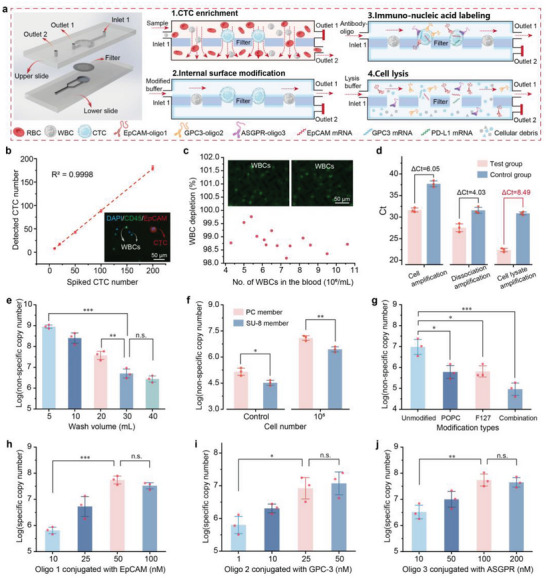
Optimization of conditions for the MRX‐CTC chip using artificial samples. a) Schematic diagram of the structure of the MRX‐CTC chip and the operational workflow for the simultaneous acquisition of multidimensional biomarkers of CTCs. b) Dynamic range of CTC recovery observed through the MRX‐CTC chip using artificial samples containing 0 to 200 CTCs. The data are presented as the means ± SDs of three independent assays. Representative merged fluorescence images of CTCs (DAPI^+^/CD45^−^/EpCAM^+^) and WBCs (DAPI^+^/CD45^+^/EpCAM^−^) captured by the chip are shown in the bottom right corner. **c)** WBC depletion rates observed with the MRX‐CTC chip using blood samples containing different numbers (4–11 × 10^6^) of leukocytes. The leukocytes retained by the chip are shown in the upper panel. d) Comparison of Ct values between the test and control groups under different oligo collection schemes (antigen‒antibody dissociation solution collection, cell lysate collection, and cell lysis solution collection). The data are presented as the means ± SDs of three independent assays. **e)** Magnitudes of nonspecific adsorption observed with different wash volumes. The data are presented as the means ± SDs of three independent assays. f) Magnitudes of nonspecific oligo adsorption observed with (10^5^ cells) or without (control) the passage of cells through the MRX‐CTC chip embedded with the PC membrane or SU‐8 membrane under a wash volume of 30 mL. The data are presented as the means ± SDs of three independent assays. g) Magnitudes of nonspecific adsorption observed after the passage of 10^4^ cells through the MRX‐CTC chip embedded with the SU‐8 membrane with different types of modifications and washed with 30 mL of wash buffer. The data are presented as the means ± SDs of three independent assays. h–j) Copy number magnitudes of specifically coupled oligos obtained via the MRX‐CTC chip under the optimal operational process at different oligo concentrations, that is, h) oligo 1 for EpCAM, i) oligo 2 for GPC‐3, and j) oligo 3 for ASGPR. The data are presented as the means ± SDs of three independent assays. The significance of differences between groups was assessed via one‐way ANOVA.

The integrated MRX‐CTC chip comprises two layers of PDMS devices and a windmill‐like hole array filter, allowing the continuous operation of CTC enrichment, inner surface modification, immunonucleic acid labeling, washing, and lysis steps, ultimately yielding quantification of protein oligos and well‐preserved mRNAs (**Figure** **3**a). The double‐layer PDMS device with radial channels for cross‐flow injection was fabricated by pouring PDMS into an aluminum mold with specific structures and then embedding the windmill‐like hole array filter into the double layers via oxygen plasma bonding (Figure S1, Supporting Information).

The microfluidic chip's architecture comprises two key components: a lower PDMS layer connected to outlet 2, which forms a filtration channel in series with inlet 1, and an upper PDMS layer linked to outlet 1, creating an incubation and washing channel (Figure 3a). The filtration membrane, situated within the lower channel, facilitates CTC capture while allowing the passage of white blood cells and lysed erythrocytes through outlet 2. Concurrently, the upper channel generates lateral shear flow, crucial for nucleic acid washing and a series of continuous operations including antibody‐oligonucleotide labeling, washing, cell lysis, and lysate collection.

A notable feature of this chip design is the incorporation of radial channels that enable cross‐flow injection. This innovation produces lateral cross‐flow, significantly enhancing sample mixing and supporting efficient nucleic acid washing. The culmination of these design elements allows for the extraction of multi‐dimensional biomarkers—both protein and mRNA—from CTCs. Consequently, this approach facilitates a comprehensive, multi‐dimensional analysis of CTC transcriptional and phenotypic profiles, offering unprecedented insights into cancer biology and potential clinical applications.

To evaluate the performance of the integrated MRX‐CTC chip, we determined the dynamic range of the CTC chips and performed statistical analysis of leukocyte depletion rates via artificial samples with different concentrations of CTCs ranging from 0 to 200 per mL of blood. We confirmed the consistency of the CTC recovery rates (y = 0.901 × −1.101, R^2^ = 0.9998) (Figure 3b) and the high leukocyte depletion rate (98.9 ± 0.4%) (Figure 3c).

### 2.5 Optimization of MRX‐CTC Chip Operation for MRT‐dPCR

The key step in the process is the collection of oligos for effective detection and protein quantification. We explored three approaches for oligo collection (antigen‒antibody dissociation solution, cell supernatant, and cell lysis solution) and assessed their viability via PCR. A test group and a control group were established, each comprising 1.0 × 10^5^ cells. The test group received a premixed solution of antibodies and oligos, whereas the control group received an equivalent concentration of premixed oligo solution. As shown in Figure 3d, the ΔCt values for the test (Ct = 22.4 ± 0.4) and control (Ct = 30.9 ± 0.4) groups amplified with the cell lysis mixture were greater than those of the other methods, with a difference of 8.49. Furthermore, both the test and control group Ct values fell within a reasonable range when the cell lysis solution collection strategy was used. This outcome suggests that extraction with cell lysis solution produces a greater yield of specific oligos, meeting the requirements for subsequent nucleic acid detection of cell surface proteins.

Furthermore, nonspecific adsorption of oligos to cells and devices can greatly affect the sensitivity of detection. To mitigate this nonspecific adsorption, parameters such as wash volume, membrane type, and chip modification were optimized. Nonspecific adsorption was then quantified through qPCR analysis of the lysate extracted from the chip via a standard equation for primer pairs (Figure S2, Supporting Information). Figure 3e illustrates the influence of wash volume on nonspecific nucleic acid adsorption, showing that volumes exceeding 30 mL had no significant effect on nonspecific adsorption. Then, with the optimal 30 mL wash volume, we evaluated nonspecific adsorption on chips with different filters (commercial PC membrane and custom SU‐8 membrane) using samples containing 0 or 10^5^ cells. The results revealed significantly lower nonspecific adsorption on the SU‐8 membrane than on the PC membrane (0 CTC: * *p* < 0.5; 10^5^ CTC: ** *p* < 0.01), which was attributed to the high porosity of the SU‐8 membrane (Figure 3f). Various modifications, including POPC, F127, and combinations of factors, have been explored to mitigate the nonspecific adsorption of nucleic acid to cells and devices. As depicted in Figure 3g, the combination of POPC and F127 modifications significantly reduced nonspecific adsorption to the chip (with the SU8 membrane) and cells by two orders of magnitude, from log7.0 ± 0.4 to log5.0 ± 0.3. In summary, to minimize background adsorption, chips should be synthesized with SU8 membranes and subjected to POPC and F127 combination modifications and washed with 30 mL of wash buffer after cell filtration, incubation with cell lysis solution, and lysate collection for subsequent detection.

### 2.6 Optimization of Antibody‒Oligo Conjugates

Drawing upon existing data pertaining to HCC, we identified surface markers that are highly expressed in HCC CTCs, HCC cell lines, and primary tumor tissues, but scarce in leukocytes. Three candidate antibodies—anti‐EpCAM, anti‐ASGPR, and anti‐GPC‐3—were selected to target these specific surface markers, aiming to achieve the desired sensitivity and specificity for HCC CTC identification. To obtain robust positive signals, experiments were conducted with varying concentrations of oligos in the experimental group (containing antibody‒oligo conjugates) and a control group (buffer solution with oligos only), and background interference was measured. The nucleic acid‐specific conjugation of 1.0 × 10^4^ cells on the chip under different oligo concentrations was assessed via qPCR. The results suggested the following optimal concentrations of oligos conjugated with each antibody (anti‐EpCAM, anti‐GPC‐3, and anti‐ASGPR): oligo 1 at 50 nm (Figure 3h), oligo 2 at 25 nm (Figure 3i), and oligo 3 at 100 nm (Figure 3j).

### 2.7 MRT‐dPCR Assay of CTC Proteins and mRNAs in Artificial Samples

**Figure 4:**
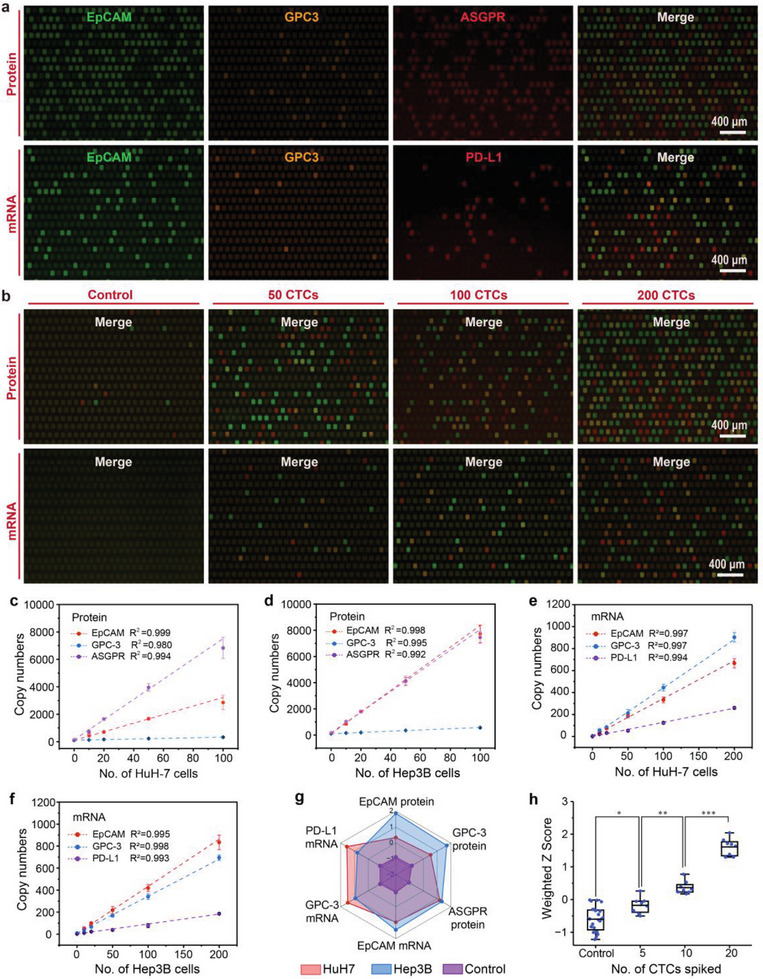
Cross‐dimensional MRT‐dPCR assay for the quantification of multidimensional CTC biomarkers in artificial samples using the MRX‐CTC chip. a) Fluorescence images of endpoint wells positive for HCC CTC proteins (EpCAM, GPC‐3, and ASGPR) and mRNAs (EpCAM, GPC‐3, and PD‐L1) in the FAM, VIC, and CY5 channels. b) Merged fluorescence images of different protein and mRNA markers observed via the MRT‐dPCR in samples containing 0, 50, 100, and 200 CTCs. c–f) Standard curves generated from MRT‐dPCR assays of six biomarkers in two types of cell lines (HuH‐7 and Hep3B). c) Standard curves for protein markers (EpCAM, GPC‐3, and ASGPR) in HuH‐7 cells across different CTC concentrations. d) Standard curves for protein markers in Hep3B cells across different CTC concentrations. e) Standard curves for mRNA markers (EpCAM, GPC‐3, and PD‐L1) in HuH‐7 cells. f) Standard curves for mRNA markers in Hep3B cells. The data are presented as the means ± SDs of three independent assays. g) Radar plot illustrating the MRT‐dPCR analysis of six biomarkers from three different cohorts (HuH7, Hep3B, and control group). HuH7 is represented by red dots, Hep3B by blue dots, and the control group by purple dots. h) Box plot representing the HCC CTC weighted Z scores for 0 CTCs (n = 16), 5 CTCs (n = 8), 10 CTCs (n = 8), and 20 CTCs (n = 8). The significance of differences between groups were assessed via one‐way ANOVA (*p < 0.05; **p < 0.01; ***p < 0.001).

To assess the feasibility of using the newly developed MRT‐dPCR for multiplex quantification analysis of proteins and mRNA markers in HCC CTCs, artificial samples containing 0–200 HuH‐7 and Hep3B cells were spiked into 1 mL of blood and subjected to MRT‐dPCR analysis following the optimized protocol. The mixture obtained from the MRX‐CTC chip, containing quantified protein oligos and well‐preserved mRNA, was injected into different regions of the digital PCR chip, enabling simultaneous compartmentalized multiplex amplification of proteins and mRNAs and real‐time fluorescence monitoring in individual microwells. As depicted in **Figure** **4**a, the distribution of endpoint wells positive for HCC CTC proteins (EpCAM, GPC‐3, and ASGPR) and mRNAs (EpCAM, GPC‐3, and PD‐L1) in the FAM, VIC, and CY5 channels was clearly demonstrated. Additionally, endpoint fluorescence images of 0, 50, 100, and 200 CTCs for both protein and mRNA in the three channels were recorded, allowing qualitative observation of the increase in the number of positive wells with increasing numbers of cells. More importantly, beyond merely obtaining fluorescence intensity data at the amplification endpoint, our multi‐channel process‐based analysis model (M‐PAM, see Notes S1 and S2, Supporting Information) allows us to capture multiplex real‐time fluorescence intensity data from over 20000 microwells throughout the entire amplification process. This enables the fitting of smooth amplification curves, facilitating a more effective classification of positive and negative curves and ultimately providing more precise results.

Figure S3a‐c (Supporting Information) illustrates the curves of simultaneous amplification in the three channels, with clear differences between the positive and negative curves. Based on these amplification curves, the fluorescence intensity addition rates and Ct values for each microwell could be calculated and tabulated (Figure S3d–I, Supporting Information). The fluorescence intensity increase rates of the positive points in the FAM (EpCAM mRNA and protein), VIC (GPC‐3 mRNA and protein), and CY5 (ASGPR protein and PD‐L1 mRNA) channels were 0.39 ± 0.07 (Figure S3d, Supporting Information), 0.66 ± 0.15 (Figure S3e, Supporting Information), and 0.25 ± 0.07 (Figure S3f, Supporting Information), respectively. The Ct values of the positive points in all three channels followed a normal distribution, with values of 26.83 ± 0.88 (Figure S3g, Supporting Information), 26.46 ± 0.86 (Figure S3h, Supporting Information), and 25.73 ± 1.21 (Figure S3I, Supporting Information). By combining correct amplification curves with fluorescence intensity addition rates and Ct values, the number of positive microwells could be accurately counted, allowing the precise calculation of copy numbers for each marker.

We summarized the marker signatures of three proteins and three mRNAs from artificial samples containing 0, 10, 20, 50, 100, and 200 CTCs (HuH‐7 and Hep3B cells) in a total of 48 cases via a heatmap (Figure S4, Supporting Information). The copy number of each target in all cases was incremented by 1 and then log2‐transformed, and the Z score was then standardized. As depicted in the heatmap, stronger signal was observed with increasing cell number, and differences in signal intensity between different cases were apparent. Additionally, we validated the correlation between marker copy numbers in different cell lines and cell counts. Figure 4c‐f presents the standard curves of the six targets in HuH‐7 and Hep3B cells against the number of cells. The protein target copy number in these cell lines exhibited a linear correlation with the cell count in the range of 0–100 cells, whereas the mRNA target copy number maintained a linear correlation within the 0–200 cell range. The detailed data for all standard curves are provided in Tables S2 and S3 (Supporting Information), and all had R^2^ values exceeding 0.980. Furthermore, we compared the expression levels of the six markers in 100–200 HuH‐7 and Hep3B cells (Figure 4g). The radar plot revealed significantly higher expression levels of EpCAM and GPC‐3 proteins in Hep3B cells than in HuH‐7 cells, with comparable ASGPR protein levels, slightly higher EpCAM mRNA levels, and slightly lower GPC‐3 mRNA and PD‐L1 mRNA levels.

To ensure the reproducibility of the entire workflow on the real‐time digital PCR platform, we conducted CTC enrichment tests on chips with samples of varying cell concentration (3 replicates per concentration) and performed real‐time digital PCR tests for the six markers (10 replicates per concentration). We assessed variability by calculating the percentage coefficient of variation (%CV) for both the CTC recovery rates and the copy numbers of the six markers. The %CV values for the chip recovery rates were calculated to be 2.50–12.50% for intra‐assay variability and 4.48% for inter‐assay variability (Table S4, Supporting Information). The %CVs of the real‐time digital PCR assays for the six markers across different CTC concentrations ranged from 1.03% to 9.39% (Table S5, Supporting Information).

To evaluate the sensitivity of our real‐time digital PCR platform in detecting the six markers, we determined the limit of blank (LOB) and limit of detection (LOD) by repeatedly measuring EpCAM, GPC‐3, and ASGPR proteins, as well as EpCAM, GPC‐3, and PD‐L1 mRNAs, in negative controls (NTCs) and with decreasing concentrations of CTC standards (10‐15 repetitions). LOB values, calculated using non‐parametric statistics from 12 replicates (Table S6, Supporting Information), were 108.3, 100.2, 192.9 copies for protein markers (EpCAM, GPC‐3, ASGPR) and 8.0, 9.0, 4.5 copies for mRNA markers (EpCAM, GPC‐3, PD‐L1), respectively. Subsequently, we tested decreasing CTC concentrations, classifying results as positive or negative based on these LOB values. Table S7 (Supporting Information) summarizes positive results across multiple MRT‐dPCR tests. Probit regression analysis yielded LOD values (at 95% confidence level) of 6.5, 9.3, and 3.2 CTCs per mL of blood for protein markers and 11.4, 10.3, and 15.1 CTCs per mL of blood for mRNA markers, respectively. Furthermore, we evaluated the comprehensive digital scoring ability of the six markers on CTCs at low concentrations (0, 5, 10, or 20 CTCs per mL of blood). The weighted Z scores of the six markers revealed significant differences between the groups (*p < 0.5; **p < 0.01; ***p < 0.001) (Figure 4h).

### 2.8 D‐SCOUT Assay for Quantification of CTC Transcriptotypes and Phenotypes in Clinical Samples

**Figure 5:**
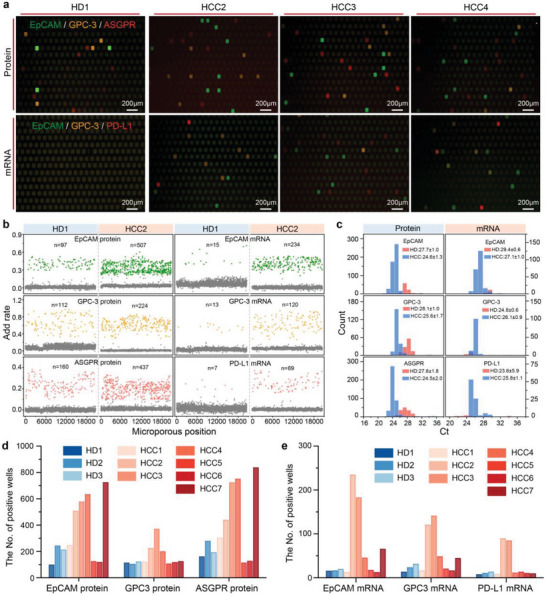
Validation of d‐SCOUT analysis for HCC CTC protein and mRNA in a small‐scale clinical trial. a) Representative merged fluorescence images of endpoint wells from d‐SCOUT assays in samples from healthy donors and HCC patients positive for protein and mRNA markers. b,c) Dynamic data for multidimensional biomarkers generated by d‐SCOUT (representative samples are shown). b) Rates of fluorescence signal increases during the MRT‐dPCR process in all microwells for each marker in the HD1 and HCC2 samples. c) Distribution of Ct values of all microwells positive for each marker in HD1 and HCC2 samples. d,e) Statistical analysis of the numbers of microwell positive for each protein and mRNA marker in d‐SCOUT assays of samples from 3 healthy donors and 7 HCC patients. The blue bars represent healthy donors, and the red bars represent HCC patients.

To assess the clinical applicability of d‐SCOUT based on MRT‐dPCR for HCC CTC transcriptome and phenotype analysis, a small‐scale clinical trial using blood samples from 3 healthy donors and 7 HCC patients was conducted. We present local multichannel fluorescence fusion images of well positive for 6 markers from one healthy donor (HD) and three HCC patients (**Figure** **5**a). Samples labeled HD1 and HCC2 were selected as representative samples, and detailed d‐SCOUT process data for each target were obtained, including the sum of fluorescence intensity rates for all wells across different channels and the distribution of Ct values for amplification curves in positive wells (Figure 5b,c). As shown in Figure 5b, the number of positive wells for the 6 markers in sample HCC2 (507, 224, 437, 234, 120, and 89, respectively) exceeded that of the healthy individuals (97, 112, 160, 15, 13, and 7, respectively). Positivity determined by fluorescence intensity addition rates was further confirmed by the narrow distribution of Ct values from the amplification curves of positive wells, as demonstrated in Figure 5c, validating the effective determination of positivity. Additionally, the number of well positive for each marker in the 10 samples is summarized in Figure 5d,e. A brief overview of target expression in the HCC samples is presented in Figure S5 (Supporting Information), which shows the expression of HCC‐related markers in 4 out of the 7 cancer patients. In conclusion, through a limited clinical trial, we achieved successful initial validation of the clinical feasibility of d‐SCOUT quantification.

**Figure 6:**
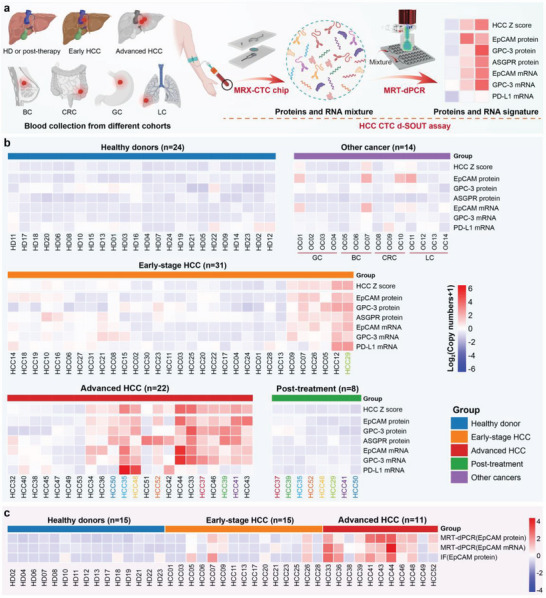
D‐SCOUT assays for simultaneous quantification of HCC CTC transcriptotypes and phenotypes across different cohorts. a) A general HCC CTC d‐SCOUT workflow developed for the simultaneous acquisition of multidimensional biomarkers via the MRX‐CTC chip, followed by accurate quantification of HCC CTC transcriptotypes and phenotypes via MRT‐dPCR. b) Heatmaps showing the signal intensities for each of the 6 HCC‐specific biomarkers and their combined expression across different patient cohorts. Blood samples were collected from 99 individuals across 5 cohorts: an early‐stage HCC cohort (n = 31), an intermediate–advanced‐stage HCC cohort (n = 22), a posttreatment HCC cohort (n = 8), an “other cancer” cohort (n = 14), and a healthy donor cohort (n = 24). Other cancers included breast cancer (BC, n = 3), colorectal cancer (CRC, n = 3), gastric cancer (GC, n = 4), and lung cancer (LC, n = 4). The colors represent individual patients before and after treatment. Primary copy numbers for each marker across all disease states were incremented by 1 and log2‐transformed. c) Heatmaps showing EpCAM signal intensities as analyzed by MRT‐dPCR or immunofluorescence across different cohorts, including HDs (n = 15), early‐stage HCC patients (n = 15), and intermediate–advanced HCC patients (n = 11). The initial results across all cohorts were incremented by 1 and then log2‐transformed. The redder the color of the tiles in the heatmap, the higher the expression of the corresponding marker in the sample; the bluer the color, the lower or absent the expression of the corresponding marker.

Next, to explore the potential of our integrated analysis platform for identifying CTC transcriptotypes and phenotypes for assessing disease progression, treatment response, and biological characteristics in HCC, we conducted a large‐scale clinical trial using d‐SCOUT quantification of CTCs. A streamlined workflow employing the optimized processing conditions for protein and mRNA profiling based on HCC CTCs and integrating MRX‐CTC chips with MRT‐dPCR for the quantification of 6 markers was developed (**Figure** **6**a). We collected blood samples from 99 individuals across 5 cohorts, namely, an early‐stage HCC cohort (n = 31), an intermediate–advanced‐stage HCC cohort (n = 22), a posttreatment HCC cohort (n = 8), an “other cancer” cohort (n = 14), and a healthy donor cohort (n = 24). HCC tumor staging was performed according to the Barcelona Clinic Liver Cancer (BCLC) staging system^[^ ^49^ ^]^ and the Chinese Staging for Liver Cancer (CNLC) guidelines. ^[^ ^50^ ^]^ Patients with Stage A or Stage I disease were included in the early‐stage HCC cohort, whereas those with Stage B‐C or Stage II‐III disease were included in the intermediate–advanced‐stage HCC cohort. The posttreatment HCC cohort included blood samples from 3 patients who had received PD‐L1 therapy and 5 patients who had received surgical resection or radiation therapy. Additionally, we investigated a cohort of patients with other cancers, including breast cancer (BC, n = 3), colorectal cancer (CRC, n = 3), gastric cancer (GC, n = 4), and lung cancer (LC, n = 4), for validation purposes. Blood samples from 24 healthy donors served as negative controls. We summarized the protein and mRNA profiles of HCC CTCs from the 99 subjects in a heatmap, complemented by weighted digital scores for the six markers, offering a comprehensive view of each sample (Figure 6b). The clinical raw data output from d‐SCOUT and the subsequent data processing procedures are detailed in the Supporting Raw Data. Notably, HCC samples presented higher signals than non‐HCC (healthy donors and other cancer types) samples did, with signals further elevated in the intermediate–advanced‐stage HCC cohort. Compared with the pretreatment samples, the posttreatment samples presented reduced signals. Interestingly, some patients in other cancer cohorts presented increased signals for EpCAM protein and EpCAM mRNA relative to those in the healthy cohort.

### 2.9 HCC CTC Z Scores for Tumor Progression

**Figure 7:**
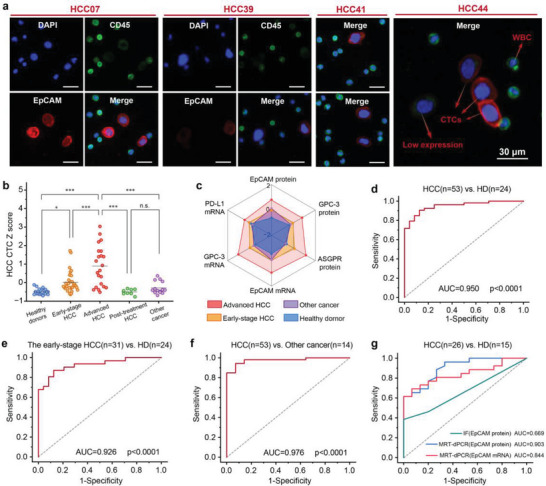
Statistical analysis of the simultaneous quantification of HCC CTC transcriptotypes and phenotypes across different cohorts via d‐SCOUT. a) Representative immunofluorescence images of CTCs captured from HCC patient samples via the MRX‐CTC chip. CTCs were identified as DAPI positive, CD45 negative, and EpCAM positive; white blood cells (WBCs) were identified as DAPI positive, CD45 positive, and EpCAM negative. Low EpCAM expression in CTCs was indicated by weak EpCAM fluorescence signal intensity. Scale bar: 30 µm. b) Scatter plot of HCC CTC Z scores across different cohorts, including the healthy donor cohort (n = 24), early‐stage HCC cohort (n = 31), intermediate–advanced stage HCC cohort (n = 22), posttreatment HCC cohort (n = 8), and other cancer cohorts (n = 14). Significant differences between groups were evaluated via one‐way ANOVA. c) Radar plot showing the Z scores of six HCC CTC biomarkers in intermediate–advanced‐stage HCC patients (red dots), early‐stage HCC patients (yellow dots), other cancers (purple dots), and healthy donors (blue dots). d–f) ROC curves of predictive probabilities from binary logistic regression analysis for the six biomarkers in d) patients with HCC versus healthy donors (HDs), e) patients with early‐stage HCC versus HDs, and f) patients with HCC versus other cancers. g) Comparison of ROC curves for distinguishing HCC patients from HD patients via different methods, including immunofluorescence for EpCAM protein and MRT‐dPCR for EpCAM protein and mRNA.

Employing a weighted Z score methodology, we computed HCC CTC Z scores for individual samples on the basis of the collective signatures of six markers. As illustrated in **Figure** **7**b, the HCC CTC Z scores in the intermediate–advanced‐stage HCC cohort were significantly greater than those in the remaining four cohorts (***p < 0.001); notably, the early‐stage HCC cohort also presented significantly higher scores than the healthy donor cohort did (*p < 0.5). Additionally, we compared the mean Z scores for each marker across the four cohorts (Figure 7c). In contrast to the healthy donor cohort, elevated Z scores for EpCAM protein and EpCAM mRNA were observed in the other cancer cohorts, whereas both the early‐stage and intermediate–advanced‐stage HCC cohorts presented varying degrees of elevation in Z scores across all the markers. Furthermore, receiver operating characteristic (ROC) analysis was conducted to evaluate the diagnostic performance of the HCC CTC Z score for distinguishing between different cohorts. The area under the ROC curve (AUC) for the multidimensional biomarker combination in distinguishing between HCC patients and healthy donors was 0.950 (95% CI, 0.897‐0.989; sensitivity = 90.6%, specificity = 87.5%, Figure 7d); detailed ROC analyses for each biomarker in discriminating between HCC patients and healthy donors are presented in Figure S6 and Table S8 (Supporting Information). Similarly, the AUC for distinguishing between early‐stage HCC patients and healthy donors was 0.926 (95% CI, 0.847‐0.982; sensitivity = 87.1%, specificity = 87.5%, Figure 7e). Moreover, the positivity of each marker in HCC patient samples was determined via ROC analysis cut‐off values against the HD cohort (Table S8, Supporting Information), and the overlapping positive counts between different markers were further quantified and depicted in a Venn diagram (Figure S7, Supporting Information). HCC samples positive for EpCAM protein, mRNA, and GPC‐3 protein and mRNA were predominantly accompanied by ASGPR protein positivity. Furthermore, several samples displayed mRNA expression without concurrent protein expression (8 cases for EpCAM and 23 cases for GPC‐3). Additionally, the ability of HCC CTC Z scores to distinguish HCC patients from those with primary malignant tumors other than HCC was explored, yielding an AUC of 0.976 (95% CI, 0.937–1.000; sensitivity = 94.3%, specificity = 92.9%, Figure 7f).

To assess the diagnostic potential of MRT‐dPCR testing and traditional CTC identification methods for HCC, concurrent MRT‐dPCR assays and immunofluorescence (IF) identification targeting the EpCAM marker on MRX‐CTC chips were subsequently conducted on 15 HD samples and 26 HCC samples. Figure 6c summarizes the EpCAM marker MRT‐dPCR assay and IF identification outcomes for 41 samples, with IF results obtained through antibody staining and manual enumeration. Representative fluorescence images of CTCs captured from patient blood samples (identified by DAPI positivity, CD45 negativity, and EpCAM positivity) are shown in Figure 7a. Finally, the discriminative ability of the MRT‐dPCR and IF methods for distinguishing between HCC patients and HDs was evaluated (Figure 7g), and the AUCs of EpCAM protein and mRNA using MRT‐dPCR were 0.903 and 0.844, respectively (sensitivities of 0.962 and 0.692, respectively), which outperformed the conventional IF identification of EpCAM protein (AUC of 0.669, sensitivity of 0.385) for CTC detection.

### 2.10 D‐SCOUT for Multidimensional Biomarker Correlation Analysis of CTCs

Using the CTC phenotypic and transcriptomic d‐SCOUT analysis platform, we conducted a correlation analysis of different biomarkers via standard samples from HCC cell lines (n = 48) and actual HCC patient samples (n = 77). Figure S8a–d (Supporting Information) displays the correlation coefficients of EpCAM protein and mRNA expression in HCC cell lines, with Spearman correlation coefficients (r_spearman_) of 0.761 and 0.581, respectively. Similarly, the corresponding coefficients for actual HCC patient samples are 0.697 and 0.436.

Furthermore, a correlation analysis among the six biomarkers in HCC patient samples was performed concurrently (Figure S8e, Supporting Information). The results revealed a strong correlation between GPC3 mRNA and EpCAM mRNA (r_spearman_ = 0.840) and a moderate correlation between the GPC3 protein and EpCAM protein (r_spearman_ = 0.533). Additionally, the ASGPR protein was moderately correlated with EpCAM mRNA (r_spearman_ = 0.662), GPC3 mRNA (r_spearman_ = 0.669), and EpCAM protein (r_spearman_ = 0.665).

### 2.11 D‐SCOUT for Digital Multidimensional Molecular Profiling of CTCs

To gain a deeper understanding of the applicability of HCC CTC d‐SCOUT technology, we further applied this strategy to 99 clinical samples, exploring the phenotypic expression patterns of CTCs across different clinical samples. Figure S9 (Supporting Information) illustrates the three‐dimensional relationship between the mRNA and protein levels of EpCAM and GPC‐3 and the HCC CTC Z scores across different cohorts. Overall, the HCC CTC Z score, along with the mRNA and protein levels of EpCAM and GPC‐3, increased with HCC progression.

**Figure 8:**
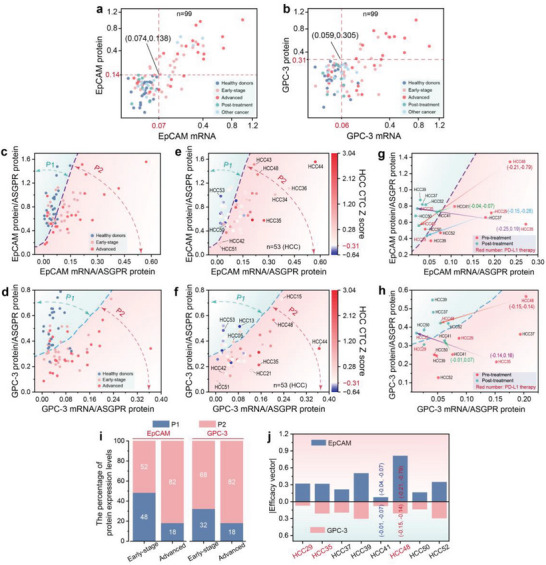
Multidimensional digital molecular information profiling of HCC CTCs via d‐SCOUT analysis. a,b) 2D distribution of EpCAM (a) and GPC‐3 (b) mRNA and protein levels across different cohorts (n = 99). The dashed lines indicate the optimal thresholds for distinguishing between healthy donor cohorts and HCC cohorts for each marker. c,d) 2D distribution of EpCAM (c) and GPC‐3 (d) mRNA and protein levels in healthy donors, early‐stage HCC patients, and middle‐to‐late‐stage HCC patients, corrected via ASGPR protein data. The dashed lines divide the cohorts into two regions: the blue region (P1) and the red region (P2). e,f 2D plots of corrected EpCAM (e) and GPC‐3 (f) mRNA and protein levels in the HCC cohort (n = 53), combined with the HCC CTC Z score. The color gradient from blue to red indicates the change in the HCC CTC Z score. g,h) Dynamic changes in corrected EpCAM (g) and GPC‐3 (h) mRNA and protein levels in HCC patients after treatment. Red labels indicate patients treated with PD‐L1 therapy, while other labels indicate patients treated with surgical resection and radiotherapy. i) Proportional stacked bar chart of EpCAM and GPC‐3 P1 and P2 in early and advanced HCC cohorts. j) Efficacy vectors of EpCAM and GPC‐3 in 8 treated HCC patients. The red numbers represent patients treated with PD‐L1 therapy.

**Figure** **8**a,b provides a more detailed view of the two‐dimensional distribution of EpCAM and GPC‐3 mRNA and protein levels across different groups. For the majority of samples from mid‐to‐late‐stage HCC patients, both EpCAM and GPC‐3 mRNA and protein levels are above the threshold (upper right quadrant). Due to the varying number of CTCs captured in each sample, it is challenging to categorize the phenotypic expression patterns of CTCs consistently across different samples. Therefore, we introduced the ASGPR protein as a variable to calibrate the protein and mRNA data of related biomarkers, resulting in a protein‒mRNA distribution at an approximately single‐cell level for each sample.

Figure 8c,d shows the distributions of EpCAM and GPC‐3 mRNA and protein levels, which were calibrated with the ASGPR protein, among early‐stage HCC patients, mid‐to‐late‐stage HCC patients, and healthy donors. Building upon this, we employed machine learning techniques to classify HD and HCC cohort. Figure S10 (Supporting Information) presents the results of 2D classification of normalized EpCAM and GPC‐3 mRNA and protein levels using Support Vector Classification (SVC) with various kernel functions (linear, poly, rbf, GSNB), and Table S9 (Supporting Information) compares the performance metrics (Accuracy, Precision, Recall, and F1) of different classifiers. The results indicate that the SVC with a polynomial kernel function (poly) demonstrated strong performance across all metrics without signs of overfitting, making it suitable for preliminary classification. The resulting classification decision boundary delineates the samples into two regions: P1 (left side), representing either the absence of CTCs or the presence of CTCs with low expression of the relevant mRNA and protein markers; and P2 (right side), indicating full expression or underexpression of the relevant marker phenotypes. Additionally, we quantified the proportions of EpCAM and GPC‐3 in P1 and P2 within early‐stage HCC and mid‐to‐late‐stage HCC cohorts (Figure 8i). The stacked bar chart reveals a progressive increase in the proportion of samples in the P2 region as HCC advances, for both EpCAM and GPC‐3.

Further, the comprehensive scores of the six HCC CTC biomarkers were mapped onto each HCC patient sample, incorporating HCC risk information derived from protein and mRNA levels to reveal the expression patterns of related biomarkers in samples with different HCC risk levels (Figure 8e,f). On the left side of the decision boundary (P1 region), the predominantly blue dots indicate HCC samples in which no CTCs were detected, such as HCC13 and HCC53. The predominantly red dots indicate HCC samples that contained CTCs but had either no or low expression of related biomarkers at both the protein and mRNA levels, such as HCC05 and HCC50. On the right side of the decision boundary (P2 region), dots closer to the boundary and positioned higher represent samples that exhibited greater consistency between protein and mRNA levels, indicating full expression of the phenotype (relatively high protein and mRNA levels), such as HCC43 and HCC48 (for EpCAM) and HCC15 and HCC48 (for GPC‐3). Points further from the boundary represent samples with poorer consistency between protein and mRNA levels, indicating underexpression of the phenotype (relatively low protein levels and relatively high mRNA levels), as seen in HCC35 and HCC44. Points near the origin signify low or undetectable expression of CTC‐associated biomarkers at both protein and mRNA levels, as exemplified by HCC42 and HCC51.

Additionally, we observed changes in EpCAM and GPC‐3 mRNA and protein levels in HCC patients before and after treatment (Figure 8g,h). As indicated by the arrows, several typical pre‐ and posttreatment samples were marked with efficacy vectors ($\vec{E}$). After treatment, the EpCAM and GPC‐3 mRNA and protein levels in HCC patients shifted toward the left side of the boundary line, particularly following PD‐L1 therapy. The significant leftward shift in EpCAM mRNA and protein levels suggests the marked therapeutic efficacy of PD‐L1 treatment (HCC29, HCC35 and HCC48). To quantify therapeutic efficacy, we analysed the direction and distance of the 2D coordinates of the related markers before and after treatment (i.e., efficacy vector $\vec{E}$) in 8 treated HCC patients. As shown in Figure 8j, the 2D changes in EpCAM appeared to be more substantial than those in GPC‐3 under the same treatment conditions. Integration of the vector changes of EpCAM and GPC‐3 revealed significant therapeutic effects in HCC48 after PD‐L1 treatment and in HCC39 after surgical resection. In contrast, the therapeutic effect of radiotherapy in HCC41 was relatively less pronounced.

## 3 Discussion

Although CTCs hold significant promise in the clinical management of HCC, current methodologies and platforms for CTC detection fall short in quantifying multidimensional biomarkers, hindering the comprehensive understanding of tumour biology, diagnosis, and prognosis. Here, we successfully developed and validated a CTC d‐SCOUT technology for simultaneous quantification of the transcriptional and phenotypic profiles of HCC CTCs. Using molecular labels and MRX‐CTC chips, intact mRNAs and quantitatively oligo‐labeled proteins were obtained, thereby streamlining the process of acquiring multidimensional molecular information from CTCs. Then, with downstream cross‐dimensional MRT‐dPCR, three validated HCC‐specific protein markers and three mRNA markers were accurately and simultaneously quantified. The resulting multidimensional digital profiling of HCC CTCs provides holistic insights into HCC progression, treatment response assessment, and biological characteristics, demonstrating significant potential in the personalized management of HCC.

One of the key hurdles in the development of a cross‐dimensional MRT‒dPCR platform for analyzing the transcriptional and phenotypic profiles of HCC CTCs is the challenge of quantifying proteins and mRNAs via a unified standard, despite their distinct molecular properties. To address this, we used immuno‐PCR^[^ ^51^, ^52^ ^]^ to enable nucleic acid‐based detection of CTC surface proteins, allowing concurrent analysis of proteins and mRNAs. We chose the streptavidin–biotin binding method for synthesizing antibody–oligo conjugates due to its cost‐effectiveness, controllability, and enhanced sensitivity. ^[^ ^53^, ^54^ ^]^ This method allows a single antibody to bind multiple oligos, improving detection sensitivity, while adjusting antibody and oligo concentrations fine‐tunes detection conditions. Our molecular labeling approach demonstrated selective binding to HCC cells, confirming its effectiveness (Figure 2b,e). When collecting oligonucleotides from labeled proteins on HCC CTCs, we tested various methods (antibody dissociation‐based collection, direct cell collection, and cell lysis solution collection), finding that cell lysis solution provided the highest sensitivity (Figure 3d). Detailed analyses were conducted to elucidate the challenges encountered with the other methods. Specifically, we found that the low efficiency of antigen‒antibody dissociation resulted in diminished sensitivity, whereas the direct cell collection method faced challenges with uneven sampling and complexities in downstream operations.

The introduction of nucleic acids presented a significant challenge, specifically regarding the issue of nonspecific nucleic acid adsorption, a common difficulty encountered in many related techniques.^[^ ^55^, ^56^, ^57^, ^58^ ^]^ Conventional methods use repetitive centrifugation and washing to remove nonspecifically bound nucleic acids, which is labor‐intensive and risks losing rare markers during sample transfer. To overcome this, we engineered an MRX‐CTC chip with a built‐in filter.^[^ ^44^ ^]^ This design incorporates radial cross‐flow and independent channels for filtration, incubation, and washing, allowing for seamless CTC enrichment, immunonucleic acid labeling, and biomarker collection without the need for centrifugation. Moreover, this approach facilitated lateral flow washing of large‐volume solutions without sample transfer, efficiently removing nonspecifically adsorbed nucleic acids. Additional surface modifications of the device were made to mitigate nonspecific nucleic acid adsorption. Our experimental findings, under optimized conditions, the MRX‐CTC chip demonstrated high CTC recovery and effective leukocyte removal, providing robust positive signals for various biomarkers (Figure 3b,c; Table S7, Supporting Information).

The most unique feature of our self‐developed platform is the cross‐dimensional MRT‐dPCR detection of CTC transcriptional and phenotypic profiles. Unlike conventional digital PCR techniques,^[^ ^1^, ^28^, ^29^, ^59^ ^]^ which rely on static analysis of endpoint reads, our previously developed RT‒dPCR platform employs a dynamic amplification process across all subsamples and uses an enhanced data classification method to deliver more precise absolute quantification outcomes.^[^ ^47^ ^]^ Nevertheless, its limited target range limits its clinical utility. To address this limitation, building on our previous investigations,^[^ ^46^, ^47^ ^]^ we expanded the number and dimensions of the targets through multiplex detection for the absolute quantification of rare HCC CTC transcriptional and phenotypic profiles, thereby offering more comprehensive and precise data for HCC clinical management. The results from testing artificial standards on the MRX‐CTC chip via MRT‐dPCR revealed exceptional reproducibility (average %CV = 1.80‐6.05%, Table S5, Supporting Information), high sensitivity (LOD as low as 3.2 CTCs per mL of blood for each marker, Table S6, Supporting Information), and robust correlations with the numbers of CTCs from different types (R^2^ ≈ 0.99, Figure 4c–f). Furthermore, our compiled data showed that the characterization outcomes of various protein markers via MRT‐dPCR results and those obtained via the use of antibody dyes for immunofluorescence were consistent (Figures 2c and 4g), further supporting the reliability of our MRT‐dPCR findings. Compared with the 2^−∆∆Ct^ qPCR relative quantification method,^[^ ^7^, ^48^ ^]^ MRT‐dPCR exhibited significantly greater differences in the absolute quantification results of CTC markers in artificial samples with fewer than 100 cells (Figures 2d and 4h; Figure S4, Supporting Information). We suggest that this discrepancy may be attributable to the laborious steps involved in traditional methods, leading to CTC loss and contamination of CTC mRNA by mRNA from a relatively large number of leukocytes, essentially obscuring CTC signal with irrelevant background. This further underscores the value of our integrated, continuous‐operation MRX‐CTC chip for cross‐dimensional MRT‐dPCR detection. Furthermore, our experimental data demonstrated that when MRT‐dPCR and immunofluorescence were simultaneously conducted on 15 HD samples and 26 HCC samples via the MRX‐CTC chip, MRT‐dPCR detection resulted in significantly better discrimination between HCC and HD than traditional immunofluorescence methods (Figure 7g). Compared with several previously reported systems, such as folate‐mediated CTC qPCR systems,^[^ ^32^, ^33^, ^34^ ^]^ CTC‐specific mRNA transcript reverse‐transcription ddPCR systems,^[^ ^1^, ^35^ ^]^ single EV surface protein ddiPCR,^[^ ^36^ ^]^ and tumor‐derived exosomal protein‐microRNA pair Exo‐PROS platforms,^[^ ^60^ ^]^ our d‐SCOUT technology offers the following advantages: (i) accurate absolute quantification and (ii) simultaneous analysis of multidimensional markers. The unprecedented exploration of d‐SCOUT detection of HCC CTC protein and mRNA holds great promise for understanding HCC progression, prognosis, and heterogeneity.

Our streamlined digital scoring system that integrates transcriptional and phenotypic profiles of HCC CTCs exhibited notable accuracy in distinguishing HCC patients, even early‐stage HCC patients, from HD patients (Figure 7d,e), underscoring its potential utility for early HCC diagnosis (sensitivity = 87.1%, specificity = 87.5%). We also observed relatively lower discriminatory performance for the GPC‐3 protein and PD‐L1 mRNA (Supplementary Figure S6), possibly because of their low expression levels, as validated in prior characterizations of HCC cell lines. The specific expression patterns of GPC‐3 protein and PD‐L1 mRNA might vary due to differences in HCC subtype, disease stage, and individual patient characteristics.^[^ ^61^ ^]^ Nevertheless, the GPC‐3 protein has emerged as a potential highly specific target for HCC,^[^ ^62^ ^]^ while ≈25% of HCC cases are found to have high levels of PD‐L1 expression,^[^ ^63^ ^]^ a typical marker for tumor immunotherapy. Despite the variability in their expression levels and differences observed across different cell lines and actual cases, we retained GPC‐3 protein and PD‐L1 mRNA as assessment indicators for HCC diagnosis and therapeutic response. Furthermore, we established a digital Z scoring system based on multidimensional biomarkers of HCC CTCs from MRT‐PCR data, effectively mitigating the potential impacts of a few suboptimal GPC‐3 protein and PD‐L1 mRNA results on the overall assessments. MRT‒PCR analysis of clinical samples from various HCC cohorts revealed substantial fluctuations in the comprehensive Z scores of the HCC CTCs throughout the progression of HCC, with individual molecular Z scores also showing varying degrees of elevation with HCC progression, providing detailed insights into HCC progression and revealing molecular heterogeneity during disease progression (Figure 7b,c). In the early‐stage HCC cohort, we observed two patients (HCC12 and HCC29) with relatively high scores for various markers and comprehensive scores, suggesting that continuous monitoring of these patients may be warranted due to an elevated risk of future HCC progression.

Our d‐SCOUT technology for HCC CTC analysis also provides comprehensive data on HCC biomarkers at both the protein and mRNA levels. This dual‐modality approach offers unique insights into tumor progression by revealing discrepancies between transcriptional and translational processes. For instance, our analysis revealed that the correlation between EpCAM and GPC‐3 at the protein and mRNA levels in actual HCC samples was lower than that in HCC cell lines (Figure S8, Supporting Information). This discrepancy highlights the dynamic nature of tumor progression, where the complex regulatory network of internal and external factors modulate HCC CTC biomarkers in vivo, including transcription factors, posttranslational controls, epigenetic modifications, and noncoding RNA‐mediated regulation.^[^ ^19^ ^]^ Notably, we observed a significant difference in protein level variations between EpCAM and GPC‐3, despite similar mRNA variations. This suggests that GPC‐3 may undergo substantial translational and posttranslational regulation, such as phosphorylation^[^ ^64^ ^]^ or ubiquitination,^[^ ^65^ ^]^ potentially exerting a greater influence on tumor progression through protein‐level regulation compared to transcriptional changes. This phenomenon warrants further investigation. Moreover, the strong correlation between GPC‐3 mRNA and EpCAM mRNA suggests their co‐expression during HCC progression, potentially indicating synchronized transcriptional regulation in advancing tumors. The moderate correlation (r_spearman_>0.66) between ASGPR protein and EpCAM mRNA, GPC‐3 mRNA, and EpCAM protein implies that combining these biomarkers could provide a more comprehensive view of tumor progression, potentially improving HCC diagnostic efficacy. Hence, our dual‐modality approach not only enhances diagnostic capabilities but also provides crucial insights into the complex interplay between transcriptional and translational processes during HCC progression, offering a more nuanced understanding of tumor biology.

Through multidimensional digital molecular information profiling of HCC CTCs combined with HCC risk scores for each sample, we can preliminarily distinguish the expression patterns of CTC‐related biomarkers to assess HCC metastatic risk (Figure 8e,f). We found that in patients at risk for HCC, EpCAM or GPC‐3 biomarkers, such as those for HCC42 and HCC51, are not always expressed, indicating individual heterogeneity among HCC patients, which necessitates a comprehensive molecular assessment. Patients with high HCC risk and underexpressed EpCAM phenotypes (such as HCC44 and HCC35) may warrant special attention, as their CTCs might transition to a more invasive mesenchymal phenotype through the EMT process, which is associated with a high risk of metastasis. Patients with moderate HCC risk and underexpressed related phenotypes (such as HCC21, HCC34, and HCC36) could be particularly likely to be missed by conventional single‐phenotype‐based CTC detection methods. Moreover, by monitoring the 2D dynamic changes in CTC‐related biomarker protein and mRNA levels from before to after treatment, we can promptly assess tumor sensitivity to therapy, providing a reliable basis for treatment plan adjustments (Figure 8g,h,j). In the follow‐up of eight HCC patients (including those treated with PD‐L1 therapy, surgical resection, and radiotherapy), we observed that the mRNA and protein levels of EpCAM and GPC‐3 shifted to the left, particularly in patients treated with PD‐L1 therapy (HCC29, HCC35, and HCC48), and the 2D distribution of EpCAM shifted significantly to the left. This suggests the initial effectiveness of the treatment within a short period (1–3 days), demonstrating the utility of multidimensional biomarker scoring of HCC CTCs in evaluating therapeutic response. However, long‐term tracking of biomarkers in patients after treatment is necessary to determine the sustained treatment efficacy, thereby assisting clinicians in tailoring personalized treatment plans for each patient.

There are several promising methods on the horizon for liquid biopsy‐based HCC detection, such as ctDNA‐based methylation^[^ ^66^ ^]^ and exosome‐based mRNA signature.^[^ ^8^ ^]^ While ctDNA methylation analysis via whole‐genome bisulfite sequencing can identify early‐stage HCC,^[^ ^66^ ^]^ its application in HCC screening may face challenges such as relatively high costs and long turnaround times. On the other hand, exosome‐based mRNA detection methods for noninvasive HCC detection also appear to hold significant potential,^[^ ^8^ ^]^ yet the absence of molecular information on phenotypes restricts our comprehensive understanding of tumors. In comparison, our d‐SCOUT technology offers a significant advancement over traditional CTC detection methods, such as ddPCR ^[^ ^1^, ^35^ ^]^ and other liquid biopsy approaches.^[^ ^16^ ^]^ Different from ddPCR, which focuses primarily on nucleic acid quantification, d‐SCOUT combines both transcriptional and protein‐level data, allowing for a more comprehensive and accurate evaluation of HCC. By incorporating both mRNA and protein expression, d‐SCOUT provides insights into HCC heterogeneity and biology, enabling earlier diagnosis, better disease monitoring, and more accurate treatment efficacy assessment. This dual‐mode analysis gives d‐SCOUT an edge in capturing the complex landscape of HCC, offering a robust tool for clinical application.

However, this study has several limitations. While we have indeed expanded the dimensions and quantity of markers for HCC CTC detection and achieved their combined quantitative assessment, there are still limitations in the number of detectable biomarkers. Other valuable HCC biomarkers, such as hexokinase 2 (HK2) ^[^ ^67^ ^]^ and cytotoxic T lymphocyte‐associated antigen‐4 (CTLA‐4),^[^ ^68^ ^]^ should be further investigation. Additionally, the sensitivity of our method may be influenced by sample preparation processes, including the quality and quantity of CTCs isolated from blood samples, which could impact detection accuracy. Optimizing sample collection and processing protocols will be critical for improving performance. Moreover, more clinical samples with gold standard results are needed to validate the clinical reliability of this method. For instance, the reliability of the method could be validated by comparing the immunohistochemistry results of late‐stage postoperative liver cancer slices from HCC patients with the preoperative CTC multitarget digital scoring results from blood samples. Furthermore, long‐term studies involving high‐risk populations are essential to fully validate this approach, especially in monitoring HCC development and recurrence. Comprehensive testing of all possible causes of HCC, alongside long‐term follow‐up observations, will be necessary to ensure broader clinical applicability.

In conclusion, we developed the CTC d‐SCOUT technology, which offers a highly sensitive, robust, and practical solution for the simultaneous and accurate quantification of protein and mRNA biomarkers from HCC CTCs. With a minimum LOD of 3.2 CTCs per mL of blood and strong reproducibility, this platform effectively distinguishes between HCC patients and those with other malignancies. Additionally, d‐SCOUT demonstrates excellent performance in identifying early‐stage HCC and enables comprehensive monitoring of cancer progression through multidimensional biomarker detection. Importantly, this technology can dynamically assess treatment efficacy, such as PD‐L1 therapy, by tracking biomarker changes over time, allowing for timely adjustments in therapeutic strategies. Moving forward, further studies will focus on expanding clinical cohorts to validate the prognostic value of d‐SCOUT biomarkers and explore its potential for broader applications in personalized cancer management. This technology has the potential to revolutionize HCC diagnosis and treatment by providing clinicians with more comprehensive molecular data to guide therapeutic decisions and improve patient outcomes.

## 4 Experimental Section

### Fabrication of MRX‐CTC Chips

The comprehensive outline of the MRX‐CTC chip is depicted in Figure 2. The chip employs a sandwich‐like design, incorporating a microfilter embedded with a windmill‐like hole array and a two‐layer polydimethylsiloxane (PDMS) microfluidic device. Our past experience^[^ ^44^ ^]^ in developing the integrated microdevice with a windmill‐like hole array unveiled that a SU‐8 membrane featuring 7 × 35 µm windmill‐like holes confers rapid and efficient CTC enrichment without blockage. Hence, the SU‐8 membrane (diameter = 13 mm) with 7 × 35 µm windmill‐like holes was integrated into two‐layer PDMS device equipped with the radical channels for cross‐flow injection by combining oxygen plasma bonding, facilitating the continuous operation of a series of steps including CTC capture, antibody oligonucleotide labeling, washing, cell lysis, and lysate collection. Following the protocol outlined in our previous study,^[^ ^69^ ^]^ fabrication of the double‐layer PDMS microfluidic device involved PDMS casting, demolding from aluminum molds, and cationic bonding. For further fabrication details, please refer to the Supporting Methods and Figure S1 (Supporting Information).

### Preparation of Oligonucleotide‐Antibody Conjugates

To prevent any similarity with human gene sequences, three distinct oligonucleotide (oligo) sequences intended for antibody labeling were derived from various plant genes. The specific oligos (oligo1, oligo2, oligo3) along with their corresponding primer and probe sequences are detailed in Table S1 (Supporting Information). These unique oligos were synthesized and biotinylated by Sangon Biotech company. Prior to the antibody‐oligo conjugation process, EpCAM‐biotin (Thermo Fisher) and biotinylated oligo1 were gently and thoroughly mixed in a PBS solution at room temperature for 5 min, utilizing varying molar ratios. Subsequently, streptavidin (Thermo Fisher) was introduced to the mixture of antibodies and oligos and incubated at room temperature for 30 min. This led to a competitive binding process, wherein EpCAM‐biotin and oligo1‐biotin vied for binding with streptavidin, ultimately forming a 100 µL EpCAM‐oligo1 conjugate complex. Similarly, GPC3‐biotin (Thermo Fisher) and ASGPR‐biotin (Thermo Fisher) were individually conjugated with oligo2 and oligo3, respectively, following the aforementioned steps. These individual antibody‐oligo conjugates were freshly prepared before utilization.

### Cell Lines Culture and Cell Spiking

Hep3B cell line was purchased from the National Collection of Authenticated Cell Cultures and cultured in Eagle's Minimum Essential Medium (MEM, Gibco) with 10% fetal bovine serum (FBS, Gibco) and 1 × Penicillin‐Streptomycin‐Glutamine (Gibco). HuH‐7 cell line was obtained from the National Collection of Authenticated Cell Cultures and maintained in Dulbecco's Modified Eagle Medium (DMEM, Gibco) containing 10% FBS and 1 × Penicillin‐Streptomycin‐Glutamine. All cell lines were grown and maintained at 37 °C with 5% CO_2_ in a humidified atmosphere. The cells were harvested with 0.25% trypsin‐EDTA (Gibco) and resuspended in PBS before use.

During the initial phases of experimental optimization, 1 × 10^4^ CTCs were mixed with a white blood cell suspension from blood to fine‐tune the parameters. To replicate the actual conditions of blood samples from cancer patients and appraise the viability of the methodology and platform, a 10 µL suspension of HuH‐7 or Hep3B cells (comprising 10, 20, 50, or 100 CTCs per 10 µL) was introduced into 1 mL of blood for testing under optimal conditions. Furthermore, the exact number of CTCs spiked into the blood was accurately controlled by averaging multiple counts on a somatic cell counting slide (Citotest; Suzhou, China).

### Cell Immunofluorescence In Vitro

Immunofluorescence experiments were performed to assess the qualitative expression of three antigens (EpCAM, GPC3, and ASGPR) in liver cancer cell lines. The experimental arrangement comprised six experimental groups, wherein each antigen was administered to Hep3B and HuH7 cell lines, and two blank groups, involving Hep3B and HuH7 cells subjected to phosphate‐buffered saline (PBS, Thermo Fisher) treatment. For fluorescence microscopy observation, Hep3B and HuH7 cells were seeded into multiple confocal petri dishes (NEST, 801002) and cultured at 37 °C for 6–12 h. Upon attaining a suitable cell density, the experiment was initiated. In the initial step, the surfaces of the cell growth dishes underwent careful washing three times using 1 mL of PBS for each wash. Subsequently, a solution of 1 mL PBS containing 4% PFA was introduced to fix the cells for 15 min, followed by a PBS rinse. After fixation, the cells were blocked using 1 mL of PBS solution containing 1% BSA for 30 min and were washed with PBS. Following this, Hep3B and HuH7 cells in separate petri dishes were incubated in the dark for 1 h with 100 µL of EpCAM‐biotin, GPC3‐biotin, ASGPR‐biotin, and PBS (blank group), respectively. Following the incubation, the cells were washed and exposed to streptavidin‐PE at room temperature in the dark for 30 min. Subsequent to three PBS washes, the expression of antigens was observed under the fluorescence microscope (ZEISS).

To validate the feasibility of antibody‐oligo conjugates, fluorescently labeled oligonucleotides were introduced for the observation of their specific coupling onto cells. EpCAM‐biotin was gently mixed with oligonucleotides (5′‐biotin, 3′‐PE) in PBS. After 5 min, this mixture was further incubated with streptavidin at room temperature in the dark for 30 min, leading to the formation of PE‐labeled EpCAM‐oligonucleotide conjugates. In the meantime, HuH7 cells, which had achieved an appropriate density in confocal culture dishes, were subjected to washing with flow cytometry staining buffer (Thermo Fisher). Subsequently, the dishes were exposed to the binding buffer (4.5 g L^−1^ glucose, 5 mm MgCl_2,_ and 10% FBS in PBS) containing PE‐labeled EpCAM‐oligonucleotide conjugates and incubated with cells at 4 °C for 45 min. During this step, salmon sperm DNA (0.1 mg ml^−1^; Sigma, USA) and yeast tRNA (0.1 mg ml^−1^; Sigma, USA) dissolved in the binding buffer were co‐incubated with the cells to hinder non‐specific binding of nucleic acids. Following the incubation, cells underwent washing with wash buffer and were subsequently observed under a fluorescence microscope.

### Participants Enrollment and Sample Collection

With ethical approval from the Ethics Advisory Committee of Huashan Hospital, Shanghai Medical College, Fudan University (2020‐1228, Nov. 2020), blood samples were procured from cancer patients and healthy donors for clinical trials. The study enrolled a total of 99 participants between June 2021 and September 2023. The cohort consisted of 53 patients with HCC categorized as stageIor A (n = 31) and stageII‐III or B‐C (n = 22), along with 14 patients having other cancer types, and 24 healthy donors (HDs). HCC patients with additional malignancies or severe mental disorders were omitted from consideration. The HCC tumor categorization adhered to the Barcelona Clinic Liver Cancer (BCLC) staging system^[^ ^49^ ^]^ and the Chinese staging for Liver Cancer (CNLC) guidelines.^[^ ^50^ ^]^ Given our primary focus on the HCC cohort, the healthy control group was intended as a preliminary baseline for comparison, hence the relatively smaller sample size. Additionally, eight HCC patients who underwent radiotherapy, surgical resection, and PD‐L1 therapy were also included in the study. To augment the scope, patients with other cancer types, including breast cancer (BC, n = 3), colorectal cancer (CRC, n = 3), gastric cancer (GC, n = 4), and lung cancer (LC, n = 4), accepted CTC assessment prior to any palliative or curative treatments. Individuals (HDs) without a history of malignancy or systemic diseases were also recruited for the clinical validation and analytical assay of the CTC phenotype and transcript test.

Venipuncture for CTC detection adhered to a standardized protocol wherein the initial 2 mL of blood was discarded to preclude potential contamination from epithelial skin cells. Subsequently, five milliliters of peripheral blood were meticulously collected from each subject into Vacutainer K_2_EDTA tubes (BD Bioscience, USA). These samples were then stored at 4 °C and processed within 12 h after collection.

### Clinical Blood Sample Processing

Prior to CTC isolation, clinical blood samples required preprocessing. ≈1–3 milliliters of blood samples were lysed for 10 min using 10 mL of lyse solution, followed by centrifugation at 400 × g for 10 min to retain the cellular pellet. The resulting sample was resuspended with 200 µL of PBS and specifically labeled with 2.5 µL of anti‐human CD45 biotin, undergoing a 30‐min incubation period. Subsequently, the sample was treated with 10 µL of streptavidin‐magnetic beads (10 mg mL^−1^) for 25 min. As a result, the processed samples, in which red blood cells were lysed and white blood cells were specifically labeled with magnetic microbeads, were then prepared for sample loading.

### MRX‐CTC Chip for Cross‐Dimensional MRT‐dPCR

A syringe containing small iron balls was inserted through a Luer adapter into the upper inlet of the microfluidic chip for injection. A magnet placed adjacent to the syringe worked in tandem with the iron balls to selectively remove a fraction of white blood cells. The device was subsequently filled with PEG solution and POPC solution in sequence, each for a 5‐min duration, to immerse and minimize non‐specific cell adsorption on the device's inner surface. Prior to sample loading, the device underwent a PBS rinse and fill. Following this, the sample was propelled through the microfilter, moving from the inlet to outlet 2, at a consistent rate facilitated by the negative pressure generated via a peristaltic pump connected to outlet 2. Upon completion of blood sample filtration, the device was washed with 2 mL of PBS from the inlet to outlet 2 and the syringe was detached.

Following microfiltration of the sample solution, the microholes and microchannels were washed and modified by flushing with a PBS solution containing PEG, POPC, or F127 from the inlet to outlet 1. Then, a pre‐prepared 200 µL mixture of antibody and oligonucleotide conjugates (EpCAM‐oligo1, GPC3‐oligo2, and ASGPR‐oligo3) was introduced from the inlet to outlet 1 and incubated at 4 °C for 60 min. A new syringe was then attached, and 5–40 mL of wash solution was introduced from the inlet to outlet 1 at a flow rate of 1 ml min^−1^ to clean cells and the device, removing non‐specifically adsorbed nucleic acids. After completion of the washing step, 100 µL of MagMAX CORE lysis solution (Thermo Fisher) was introduced from the inlet, incubated for 5 min, and then the lysate was collected.

The 100 µL lysis buffer contained intact mRNA and oligonucleotides for protein quantification. Ten microliters of the buffer were diluted 1000‐fold for MRT‐dPCR protein analysis. The remaining 90 µL underwent RNA extraction using RNA‐easy Isolation Reagent (Vazyme, China) according to the manufacturer's instructions. Subsequently, cDNA synthesis was conducted using the Maxima H Minus Reverse Transcriptase Kit (Thermo Fisher) following the manufacturer's instructions. Furthermore, using DNA Digital PCR Master Mix (Thermo Fisher) as instructed, the pre‐mixed solutions containing multiplex primer‐probe systems for protein and mRNA, respectively, were prepared. The diluted lysis buffer and cDNA were separately added to the pre‐mixed solutions to prepare the MRT‐dPCR reaction mixtures. Finally, these mixtures were injected into a MAP plate (Thermo Fisher) for subsequent cross‐dimensional MRT‐dPCR assay.

### MRT‐dPCR Data Acquisition and Processing

Our self‐developed MRT‐dPCR platform comprised the air‐driven sample loading system, the temperature control system, and the multi‐channel fluorescence signal acquisition system. The MAP plate underwent 40 temperature cycles, with fluorescence images captured at the end of each cycle in three channels: FAM, VIC, and CY5. Fluorescence signals from each microwell in each channel were extracted from every image, and smoothed amplification curves for each microwell were generated using M‐PAM (Notes S1 and S2, Supporting Information). Unlike traditional endpoint analysis for positivity determination, our MRT‐dPCR system assessed microwell positivity based on individual microwell amplification curves, combined with individual microwell Ct values and fluorescence growth rates. Finally, the concentration of nucleic acids in the sample was calculated using a Poisson distribution Equation (1):

(1) $$X=-\ln\left(1-M/N\right)\times N$$

where X represents the copy number of nucleic acids in the sample, M denotes the number of positive reaction units, and N signifies the total reaction units.

### Statistical Analysis

The use of Support Vector Classification (SVC) algorithms with different kernel functions in machine learning is described in Note S3 (Supporting Information). The recovery rates of CTCs were presented as means ± standard deviations. Significant differences among various groups were assessed using one‐way analysis of variance (one‐way ANOVA). Heatmap analysis was performed using the Heatmap plot tool in Hiplot Pro (https://hiplot.com.cn/), a comprehensive web service for biomedical data analysis and visualization. LOD for each target were calculated through Probit regression analysis using SPSS Statistics software. Z‐scores were calculated based on the expression levels of three proteins and three mRNAs in Excel. The weights for each target were determined using the Analytic Hierarchy Process (AHP) method. The HCC CTC Z‐scores reflecting the progression of HCC were calculated as the sum of the weighted Z‐scores for six targets in each sample. ROC curves were generated using Origin2023 software to evaluate the diagnostic performance of each indicator. Binary logistic regression analysis was conducted using SPSS Statistics software to predict the probability of multi‐target joint diagnosis.

## Conflict of Interest

The authors declare no conflict of interest.

## Author Contributions

H.L. and J.L. are Co‐first authors. H.L. was the primary contributor to this manuscript, responsible for conceptualization, methodology, data curation, formal analysis, writing the original draft, visualization, and wrote – reviewed & edited. J.L. contributed to conceptualization and methodology. Z.Z., H.D., Q.D., Z.G., J.Y., S.L., N.P., and C.L. provided resources. Q.Y. and D.L. contributed to software development. W.Z. and L.Z. were involved in funding acquisition, methodology, and wrote – reviewed & edited.

## Supporting information



Supporting Information

Supporting Information

## Data Availability

The data that support the findings of this study are available in the supplementary material of this article.
