# Supplementary material for: Digital Quantitative Detection for Heterogeneous Protein and mRNA Expression Patterns in Circulating Tumor Cells
Source: Adv Sci (Weinh). 2024 Nov 18;12(2):2410120. doi: 10.1002/advs.202410120 (PMC11727120; doi:10.1002/advs.202410120)
Supplement: Supplementary file 1 — Supporting Information [file ADVS-12-2410120-s001.docx]

Supplementary Information for

**Digital Quantitative Detection for Heterogeneous Protein and mRNA Expression Patterns in Circulating Tumor Cells**

Hao Li^1,2^, Jinze Li^1^, Zhiqi Zhang^1^, Qi Yang^1^, Hong Du^3^, Qiongzhu Dong^4^, Zhen Guo^1,2^, Jia Yao^1^, Shuli Li^1^, Dongshu Li^1,2^, Nannan Pang^1^, **Chuanyu Li^1,2^, Wei Zhang^1,2^, Lianqun Zhou^1^**

1. Suzhou Institute of Biomedical Engineering and Technology, Chinese Academy of Science, Suzhou 215163, China

2. School of Biomedical Engineering (Suzhou), Division of Life Sciences and Medicine, University of Science and Technology of China, Hefei 230026, China

3. Department of Clinical Laboratory, The Second Affiliated Hospital of Soochow University, Suzhou 215000, China

4. Department of General Surgery, Huashan Hospital & Cancer Metastasis Institute, Fudan University, Shanghai 200040, China

## The follow sections include:

Supplementary Methods

Supplementary Figures 1 to 10

Supplementary Tables 1 to 9

Supplementary Notes 1 to 3

## Supplementary Methods

### Fabrication of MRX-CTC chips

Conforming to the protocols published in our prior study^1^, the fabrication of the two-layer PDMS microfluidic device involved demolding from aluminum molds with distinctive patterns. The mold design, created with Solidworks, was translated into reality via a 3-axis computer numerical controlled (CNC) milling machine (VA4, TSUGAMI). Following successive rinses in acetone, ethanol, and ultrapure water using an ultrasonic cleaner for 10 minutes, the molds, including both the cover layer and bottom layer molds, were dried with nitrogen. Subsequently, the molds underwent a 12-hour immersion in a 7:3 solution of ammonia to hydrogen peroxide, resulting in the hydrophilization of the mold surface. After cleaning, the molds were immersed in a 1% solution of heptadecafluoro-1,1,2,2-tetradecyl silane in n-hexane for 30 minutes, then baked on a 90 ℃ hot plate for 30 minutes to ensure hydrophobicity, aiding the release of PDMS (Sylgard 184, DOWSIL) from the molds. Following this, 5-6 g of degassed 10:1 (monomer: curing agent) PDMS was poured onto the molds with a glass slide placed atop the PDMS in the bottom layer mold. After mold baking at 100 °C for 1 hour, the PDMS was gently detached, and inlet and outlet holes were punctured. The fabrication process of the PDMS layer and microfilter with a windmill-like hole array is briefly illustrated in Supplementary Fig. 1. Ultimately, the two layers and microfilter were bonded using oxygen plasma (YZD08-5CS, SAOT Tech Co., Ltd.) at a pressure of 0.01 atm, oxygen flow rate of 0.1 ml/min, and 120W RF power for 2 minutes.

### MRX-CTC chip for immunofluorescence

A syringe containing small iron balls was inserted through a Luer adapter into the upper inlet of the microfluidic chip for injection. A magnet placed adjacent to the syringe worked in tandem with the iron balls to selectively remove a fraction of white blood cells. The device was subsequently filled with PEG solution and POPC solution in sequence, each for a 5-minute duration, to immerse and minimize non-specific cell adsorption on the device's inner surface. Prior to sample loading, the device underwent a PBS rinse and fill. Following this, the sample was propelled through the microfilter, moving from the inlet to outlet 2, at a consistent rate facilitated by the negative pressure generated via a peristaltic pump connected to outlet 2. Upon completion of blood sample filtration, the device was washed with 2 mL of PBS from the inlet to outlet 2 and the syringe was detached.

In the subsequent steps of cell immunofluorescence, 200 μL of 0.2% Triton X-100 in PBS was introduced to the CTC chip and incubated for 5 minutes to permeabilize cells. Subsequently, 200 μL of PBS was introduced at a flow rate of 50 μL/min to rinse the sample. Likewise, the sample on the membrane was subjected to a 5-minute incubation with 200 μL of 1% BSA in PBS to block nonspecific binding, followed by rinsing with 200 μL of PBS at a flow rate of 50 μL/min. The cells on the membrane were subsequently exposed to 200 μL of immunostaining solution, containing anti-EpCAM PE (1:40 dilution) and anti-human CD45 FITC (1:80 dilution), at 4 °C in the dark for 30 minutes. After this, a 200 μL rinse with PBS at a flow rate of 50 μL/min was performed. Following staining with 1 μg/mL DAPI for 8 minutes, the CTC chip underwent two rinse cycles with 200 μL of PBS at 50 μL/min, once from inlet to outlet 1 and once from inlet 1 to outlet 2, to ensure clearer observation.

## Supplementary Figures


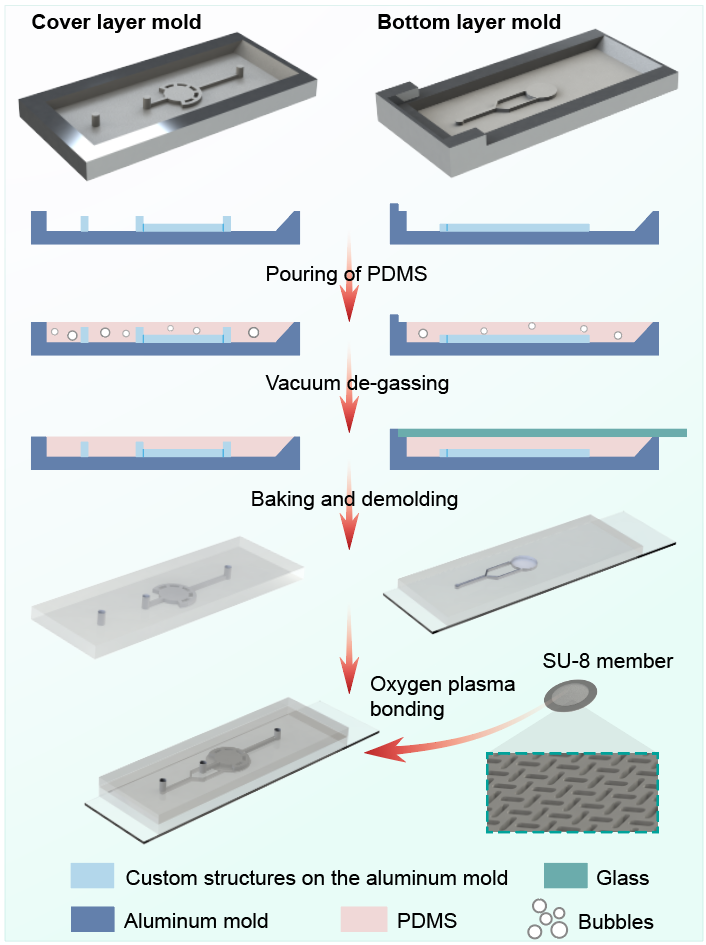


**Supplementary Fig. 1 Fabrication process of the MRX-CTC chip integrated with an SU-8 membrane.** PDMS was poured into an aluminum mold with a specific structure, followed by vacuum degassing and high-temperature baking. After demolding, a double-layer PDMS structure with radial channels for cross-flow injection was formed. Finally, an SU-8 membrane with a windmill-like pore array was embedded into the double-layer PDMS through oxygen plasma bonding to form the MRX-CTC chip.

**
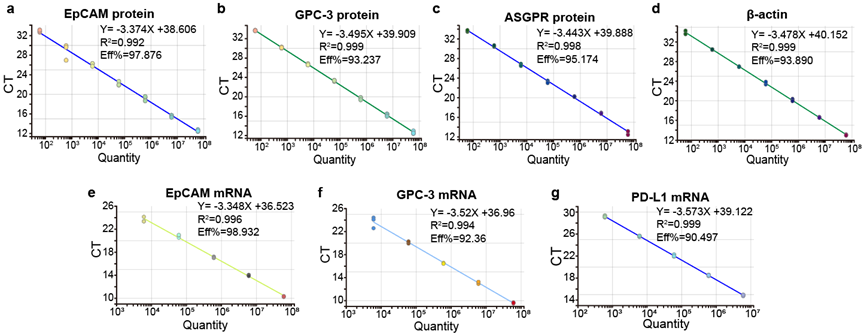
**

**Supplementary Fig. 2 Standard Curves for Gene Sequence Amplification Primers.** Standard curves were generated for the amplification primers targeting molecular tag sequences for protein markers (**a-c**), reference gene sequence (**d**), and HCC mRNA markers (**e-g**). All templates were initially diluted from a concentration of 6.022×10^7^ copies/μL in tenfold serial dilutions, with each concentration measured in triplicate. Panels **a-d** depicted six concentration gradients, while panels **e-g** depicted four concentration gradients.


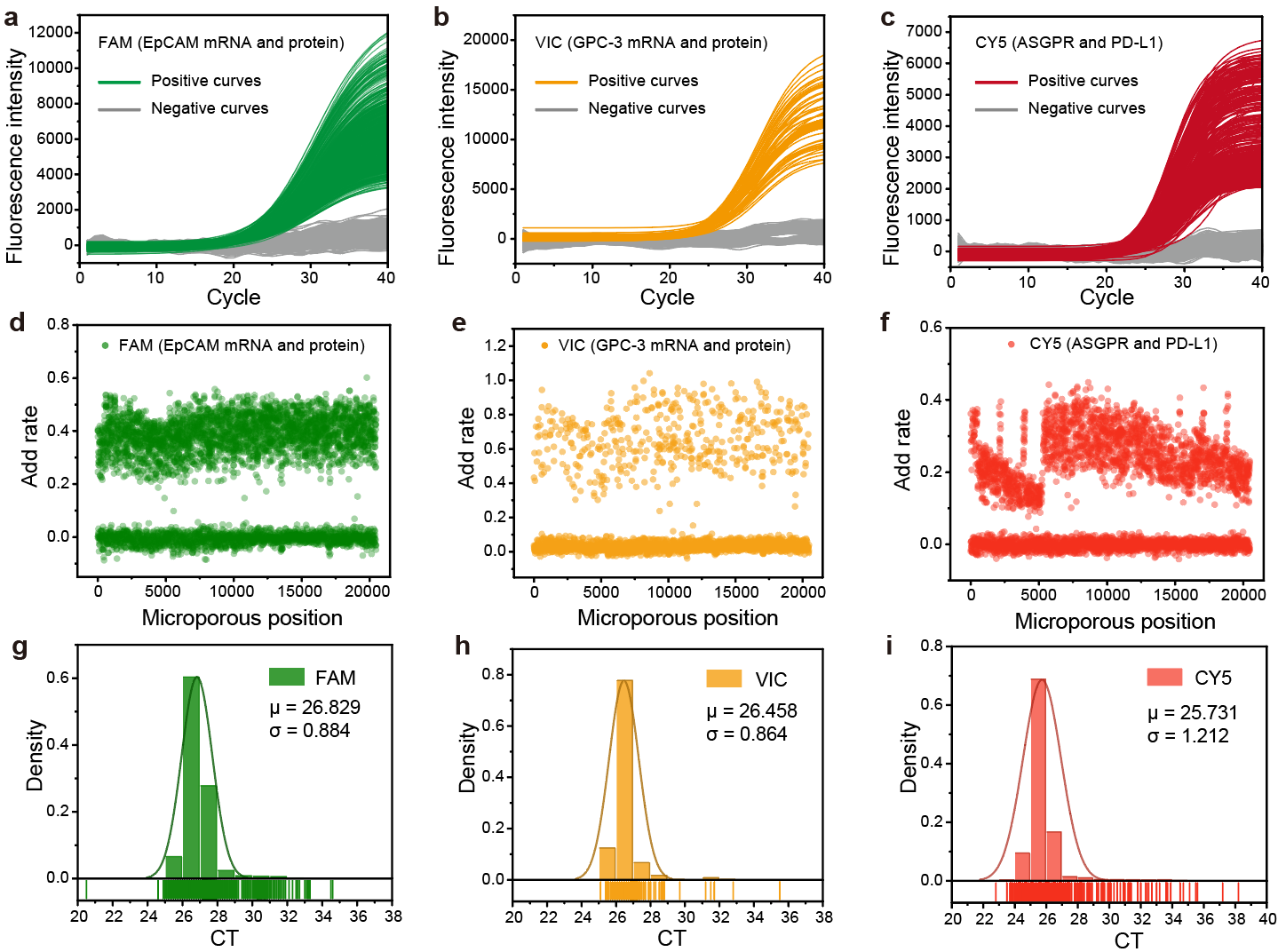


**Supplementary Fig. 3 Representative Multi-Dimensional Dynamic Data from the MRT-dPCR Process Across All microwells. a-c** The real-time amplification curves for all microwells over 40 cycles in the FAM (**a**), VIC (**b**), and CY5 (**c**) channels. **d-f** The growth rates of fluorescence intensity relative to the initial fluorescence for all microwells in the FAM (**d**), VIC (**e**), and CY5 (**f**) channels. g-i The distribution of Ct values with amplification curves for all microwells in the FAM (**g**), VIC (**h**), and CY5 (**i**) channels. EpCAM protein and mRNA were detected in the FAM channel, GPC-3 protein and mRNA were detected in the VIC channel, and ASGPR protein and PD-L1 mRNA were detected in the CY5 channel.


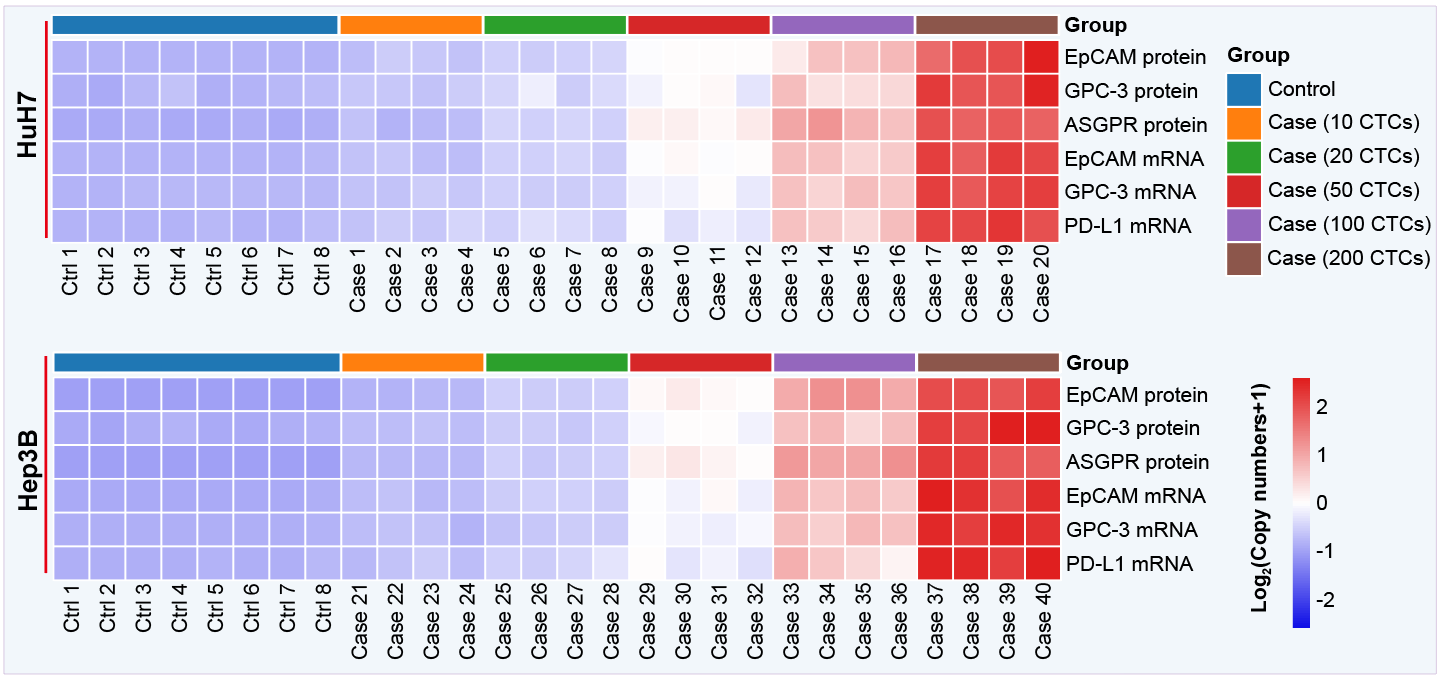


**Supplementary Fig. 4 Heatmap analysis of HCC CTC multi-biomarkers expression.** Heatmaps illustrates the expression levels of six HCC-specific biomarkers (EpCAM protein, GPC-3 protein, ASGPR protein, EpCAM mRNA, GPC-3 mRNA, and PD-L1 mRNA) in two cell lines: HuH7 and Hep3B. All groups include control samples (Ctrl 1 to Ctrl 8) and case samples (Case 1 to Case 40) with varying numbers of CTCs (10, 20, 50, 100, and 200 CTCs). Initial copy numbers were log2-transformed after adding 1 across all groups.


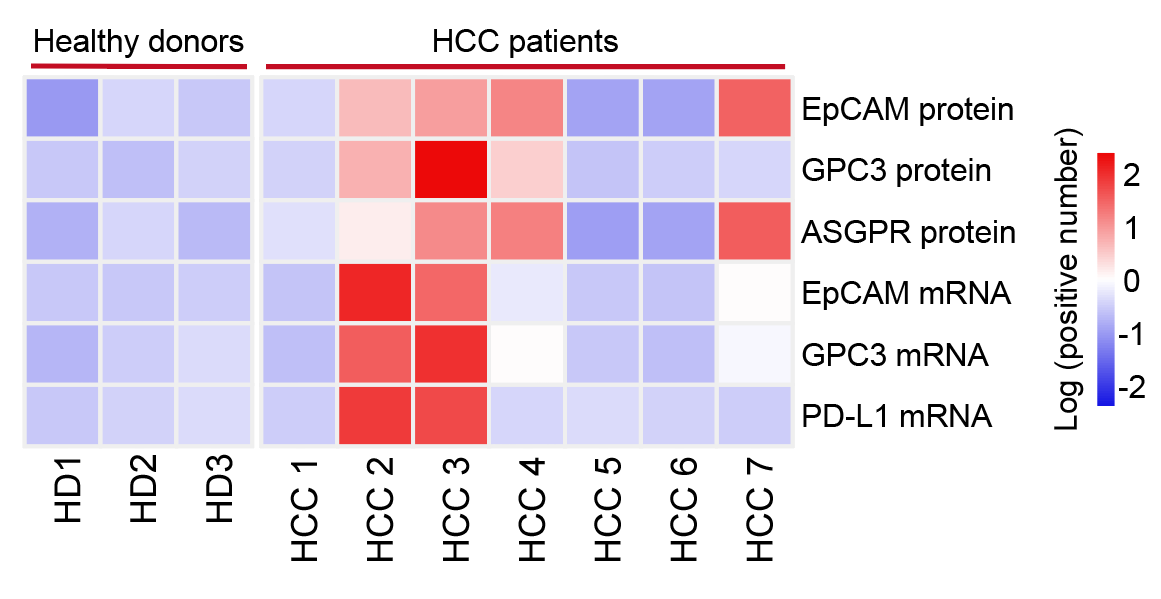


**Supplementary Fig. 5 Heatmap of biomarker expression in healthy donors and HCC patients.** This heatmap illustrates the expression levels of six biomarkers (EpCAM protein, GPC3 protein, ASGPR protein, EpCAM mRNA, GPC3 mRNA, and PD-L1 mRNA) in samples from three healthy donors (HD1, HD2, HD3) and seven HCC patients (HCC 1 to HCC 7). Initial results were log2-transformed across all samples.


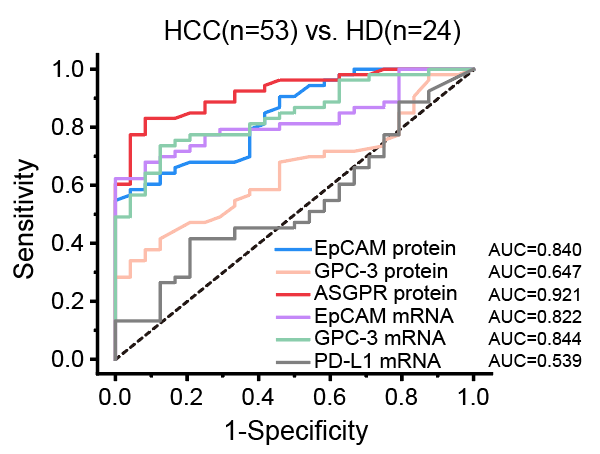


**Supplementary Fig. 6 Comparison of ROC Curves for Different Markers Distinguishing HCC Patients (n=53) from HDs (n=24).** The AUC values for EpCAM protein, GPC3 protein, ASGPR protein, EpCAM mRNA, GPC3 mRNA, and PD-L1 mRNA were 0.840, 0.647, 0.921, 0.822, 0.844, and 0.539, respectively. More detailed results of ROC curves for each markers differentiating HD and HCC are summarized in **Supplementary Table 8**. HD, Healthy Donor; AUC, area under the curve; ROC, receiver operator characteristic.


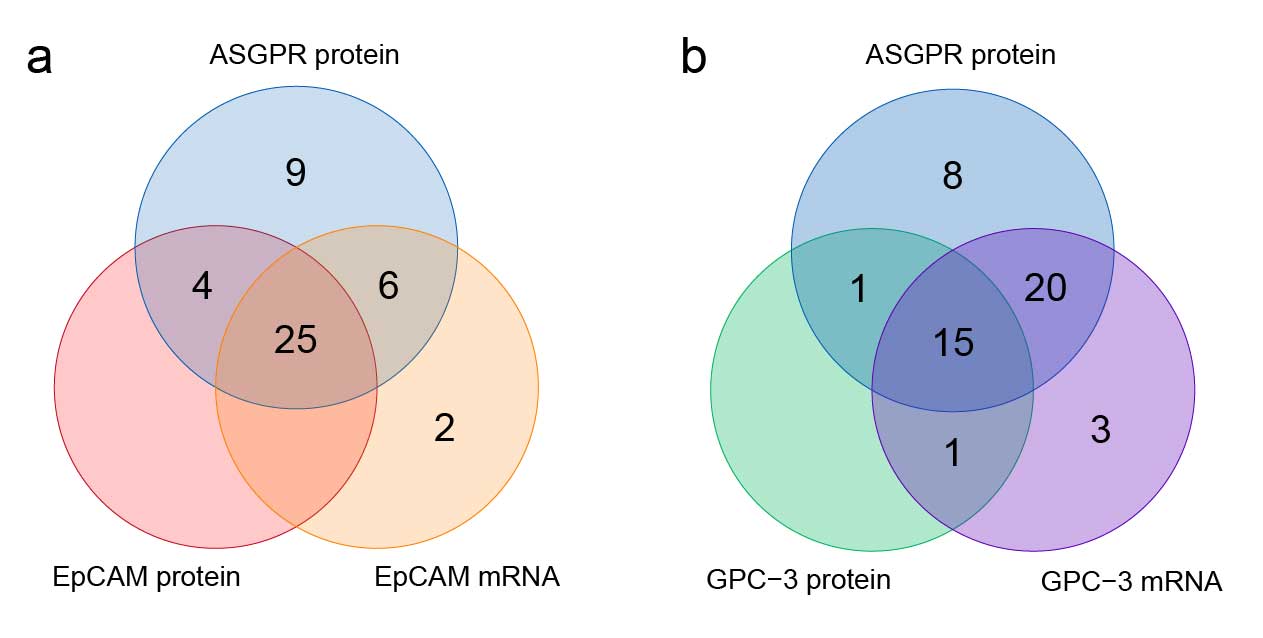


**Supplementary Fig. 7 Venn Diagrams Showing the Overlap of Biomarkers in HCC CTCs.** **a** Overlap of detected biomarkers among ASGPR protein, EpCAM protein, and EpCAM mRNA. The diagram shows the distribution of samples positive for each marker, with 25 samples exhibiting all three markers, indicating a high degree of co-expression. **b** Overlap of detected biomarkers among ASGPR protein, GPC-3 protein, and GPC-3 mRNA. The diagram shows the distribution of samples positive for each marker, with 15 samples exhibiting all three markers, indicating co-expression of protein and mRNA biomarkers.


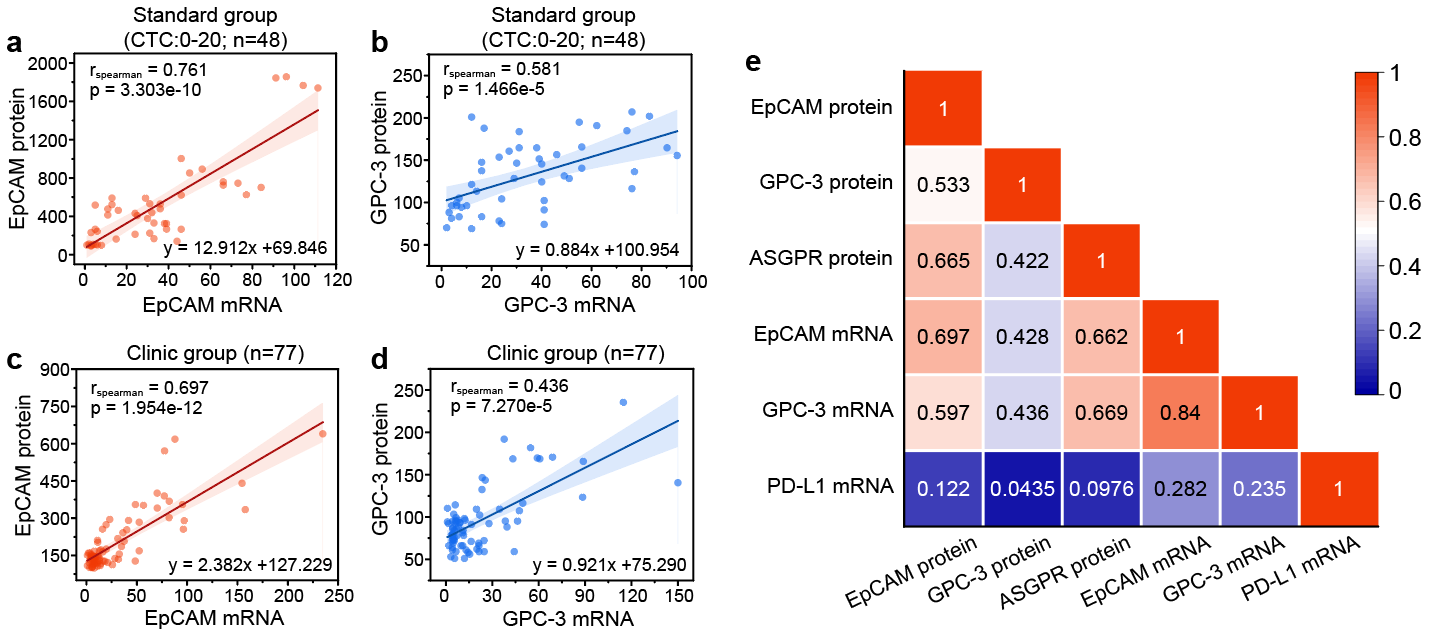


**Supplementary Fig. 8 Spearman correlation analysis of proteins and mRNA using standard and clinical samples.** **a** Spearman correlation analysis of EpCAM protein and mRNA in 48 standard samples spiked with 0-20 CTCs. **b** Spearman correlation analysis of GPC-3 protein and mRNA in 48 standard samples spiked with 0-20 CTCs. **c** Spearman correlation analysis of EpCAM protein and mRNA in 77 HCC clinical samples. **d** Spearman correlation analysis of GPC-3 protein and mRNA in 77 HCC clinical samples. **e** Heatmap of correlations between different biomarkers in clinical HCC samples. Correlation analysis was conducted using Spearman rank correlation analysis. An rSpearman value between 0 and 0.3 indicated no correlation; between 0.3 and 0.5 indicated a weak correlation; between 0.5 and 0.8 indicated a moderate correlation; and between 0.8 and 1 indicated a strong correlation.


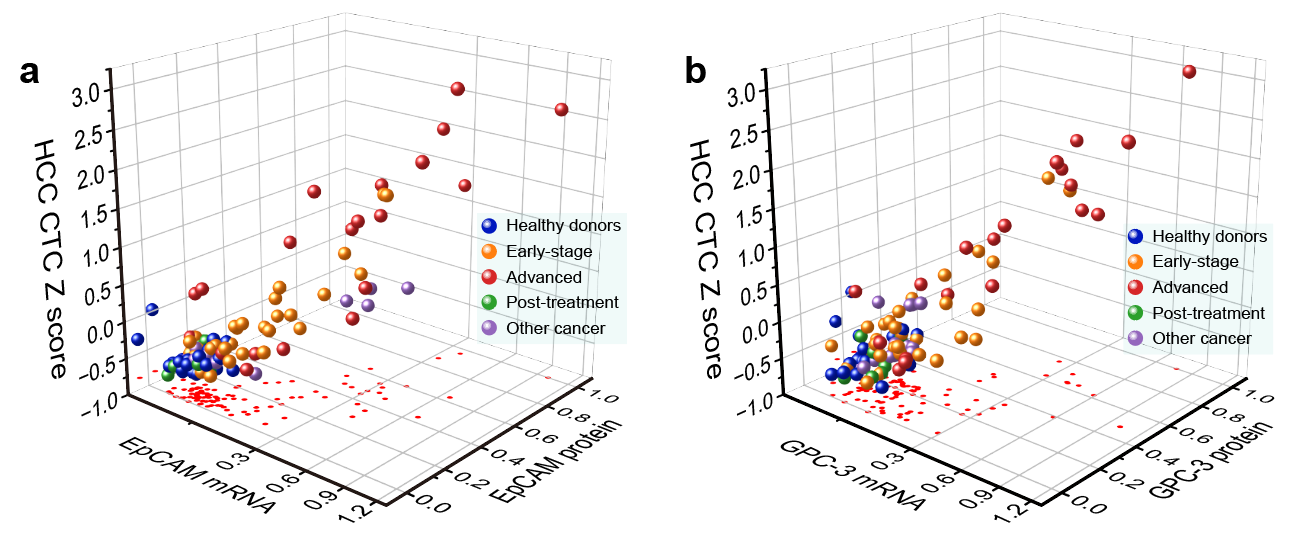


**Supplementary Fig. 9 The three-dimensional relationship between the mRNA and protein levels of EpCAM and GPC-3, and the HCC CTC Z-score across different cohorts. a** Three-dimensional distribution of EpCAM mRNA, EpCAM protein, and HCC CTC Z-scores across different cohorts, including healthy donors, early-stage HCC patients, mid-to-late-stage HCC patients, post-treatment patients, and other cancer groups. **b** Three-dimensional distribution of GPC-3 mRNA, GPC-3 protein, and HCC CTC Z-scores across different cohorts, including healthy donors, early-stage HCC patients, mid-to-late-stage HCC patients, post-treatment patients, and other cancer groups.


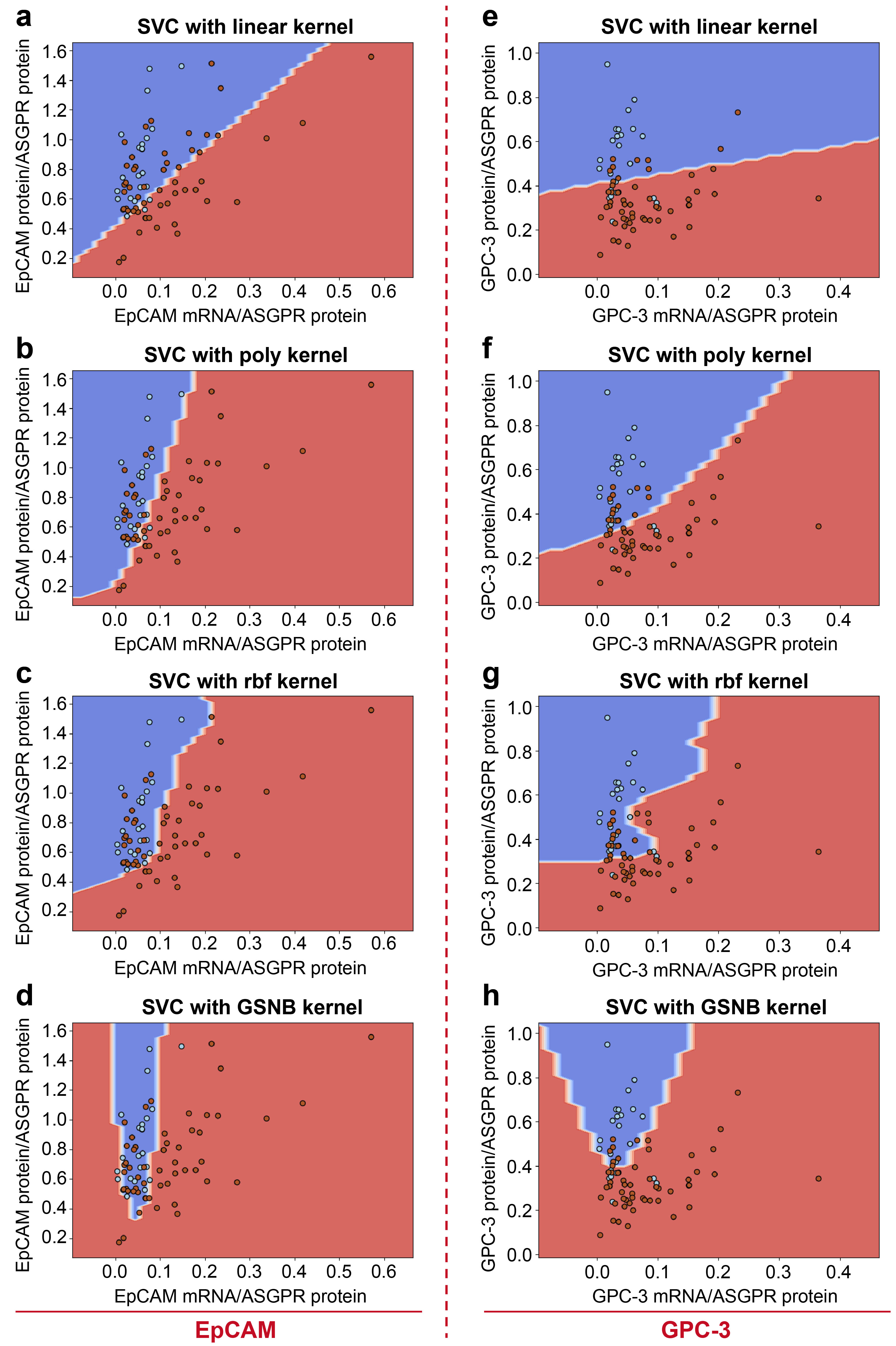


**Supplementary Fig. 10 Two-dimensional classification of normalized EpCAM and GPC-3 protein and mRNA using Support Vector Classification (SVC) with different kernels.** Panels (**a-d**) illustrate decision boundaries generated by SVCs using various kernels: linear kernel (**a**), polynomial kernel (**b**), radial basis function (RBF) kernel (**c**), and gaussian naive bayes (GSNB) kernel (**d**) for the normalized EpCAM protein and mRNA data. Panels (**e-h**) show decision boundaries generated by SVCs with linear kernel (**e**), polynomial kernel (**f**), radial basis function (RBF) kernel (**g**), and gaussian naive bayes (GSNB) kernel (**h**) for the normalized GPC-3 protein and mRNA data. Blue points represent HD samples, and red points represent HCC samples.

## Supplementary Tables

**Supplementary Table 1. Sequence of tags, primers or probes**

| The names of tags, primers or probes | Sequence (5' to 3') |
| --- | --- |
| oligo1 (for EpCAM protein) | CAAAGACTCCAGGTCCGTTAGTTTTTGACCCGTCGCTCAGCCGCTCATAACAACGAACTACAAAAGCGAGCATTTCAAAACCCGGGCATAACCTGGACACGAACACCAAC |
| Oligo1-F | GACTCCAGGTCCGTTAGT |
| oligo1-R | GTGTTCGTGTCCAGGTTAT |
| oligo1-P | FAM-TCGTTGTTATGAGCGGCTGAGCG-BHQ1 |
| oligo2 (for GPC-3 protein) | GTAACAGGTAGTAGTACGTCTGTTTCACGATGAGACAAGTCACCAATTCCATCTGAGCGTCAGTGTAGCATACTGACTCCCAGCCGATAGATAATACGAGACGAACACGT |
| oligo2-F | CAGGTAGTAGTACGTCTGTTTCAC |
| oligo2-R | GTTCGTCTCGTATTATCTATCGGC |
| oligo2-P | VIC-TCCATCTGAGCGTCAGTGTAGCAT-BHQ1 |
| oligo3 (for ASGPR protein) | CACGCAACTTCGGCATCACAGGTGTCGGAATTCCTAGTAGTACTATCACCGACGCTCAGACTATGGACGAGTTCCTCTCTCGTAAACGCTCTGCTAATACAATGGACTTG |
| oligo3-F | AACTTCGGCATCACAGGT |
| oligo3-R | CCATTGTATTAGCAGAGCGTTTA |
| oligo3-P | CY5-ACTCGTCCATAGTCTGAGCGTCG-BHQ3 |
| EpCAM mRNA-F | CGTCAATGCCAGTGTACTTCA |
| EpCAM mRNA-R | TTTCTGCCTTCATCACCAAA |
| EpCAM mRNA-P | FAM-TGTCATTTGCTCAAAGCTGGCTGC-BHQ1 |
| GPC-3 mRNA-F | AATGAAGGGCCCTGAGC |
| GPC-3 mRNA-R | GCCAGTTCTGCAAGGAAGC |
| GPC-3 mRNA-P | VIC-AGCACATTAACCAGCTCCTGAGAAC-BHQ1 |
| PD-L1 mRNA-F | CATGGACCAAGGGATTCAAG |
| PD-L1 mRNA-R | CGTCTCCTCCAAATGTGTATC |
| PD-L1 mRNA-P | CY5-ACCATACAGCTGAATTGGTCATCCC-BHQ3 |
| β-actin-F | GCCAACACAGTGCTGTCTG |
| β-actin-R | CACATCTGCTGGAAGGTGG |
| β-actin-P | ROX-TGATCTTGATCTTCATTGTGC-BHQ2 |

**Supplementary Table 2.** **Linear fitting of copy numbers versus HuH-7 CTCs number**

| Linear fitting | | | | | | |
| --- | --- | --- | --- | --- | --- | --- |
| Function equation | $y =a+bx$ | | | | | |
| CTC type | HuH-7 | | | | | |
| Targets | EpCAM protein | GPC-3 protein | ASGPR protein | EpCAM mRNA | GPC-3 mRNA | PD-L1 mRNA |
| $a$ | 98.88 | 104.31 | 136.55 | 1.33 | 3.95 | 0.29 |
| $b$ | 31.26 | 2.29 | 73.73 | 3.44 | 4.40 | 1.28 |
| $R^{2}$ | 0.999 | 0.980 | 0.994 | 0.997 | 0.997 | 0.994 |

**Supplementary Table 3.** **Linear fitting of copy numbers versus Hep3B CTCs number**

| Linear fitting | | | | | | |
| --- | --- | --- | --- | --- | --- | --- |
| Function equation | $y =a+bx$ | | | | | |
| CTC type | Hep3B | | | | | |
| Targets | EpCAM protein | GPC-3 protein | ASGPR protein | EpCAM mRNA | GPC-3 mRNA | PD-L1 mRNA |
| $a$ | 103.12 | 100.67 | 177.67 | 5.22 | 7.51 | 2.14 |
| $b$ | 82.62 | 4.77 | 78.73 | 4.29 | 3.37 | 0.90 |
| $R^{2}$ | 0.998 | 0.995 | 0.992 | 0.995 | 0.998 | 0.993 |

**Supplementary Table 4. The results of reproducibility study of MRX-CTC Chips using artificial samples**

| Run Number | No. of CTCs | Recovery rates (%) | | | Mean | SD | Intra CV (%) |
| --- | --- | --- | --- | --- | --- | --- | --- |
|  |  | test 1 | test 2 | test 3 |  |  |  |
| 1 | 10 | 0.80 | 0.70 | 0.90 | 0.80 | 0.10 | 12.50 |
| 2 | 20 | 0.75 | 0.95 | 0.90 | 0.87 | 0.10 | 12.01 |
| 3 | 50 | 0.80 | 0.86 | 0.90 | 0.85 | 0.05 | 5.90 |
| 4 | 100 | 0.87 | 0.86 | 0.92 | 0.88 | 0.03 | 3.64 |
| 5 | 200 | 0.93 | 0.90 | 0.88 | 0.90 | 0.02 | 2.50 |
|  | Inter CV (%) = 4.48 | | | | | | |

The reproducibility of MRX-CTC Chips was assessed by determining the percent coefficient of variation (%CV) for recovery rates. Intra-assay variability was evaluated by a single operator conducting three tests in one day, while inter-assay variability was assessed across three operators conducting a total of five assay runs (one run per day), with each run comprising three tests (totaling 15 chips).

**Supplementary Table 5. The results of reproducibility study of each biomarker based on MRT-dPCR using artificial samples**

| Targets | No. of CTCs | Test 1 | Test 2 | Test 3 | Test 4 | Test 5 | Test 6 | Test 7 | Test 8 | Test 9 | Test 10 | Mean | SD | %CV |
| --- | --- | --- | --- | --- | --- | --- | --- | --- | --- | --- | --- | --- | --- | --- |
| EpCAM protein | 100 | 3027.2 | 2872.6 | 2978.1 | 2945.0 | 3011.3 | 2960.9 | 2908.1 | 3089.9 | 2920.0 | 3075.2 | 2978.8 | 71.67 | 2.41 |
|  | 50 | 1366.4 | 1281.2 | 1407.6 | 1424.7 | 1388.1 | 1363.0 | 1367.6 | 1328.8 | 1380.1 | 1409.8 | 1371.7 | 42.21 | 3.08 |
|  | 25 | 626.7 | 633.1 | 618.2 | 621.4 | 631.0 | 615.0 | 633.1 | 631.0 | 621.4 | 623.5 | 625.4 | 6.47 | 1.03 |
| GPC3 protein | 100 | 1148.6 | 1033.0 | 1044.1 | 1008.8 | 1131.8 | 1026.4 | 1121.8 | 1035.3 | 1039.7 | 1085.1 | 1067.5 | 50.18 | 4.70 |
|  | 50 | 621.4 | 626.7 | 610.8 | 631.0 | 632.0 | 631.0 | 608.7 | 637.3 | 622.5 | 608.7 | 623.0 | 10.49 | 1.68 |
|  | 25 | 309.6 | 307.6 | 305.5 | 292.1 | 297.3 | 303.4 | 291.1 | 289.0 | 304.5 | 302.4 | 300.3 | 7.35 | 2.45 |
| ASGPR protein | 100 | 5185.4 | 4973.0 | 5106.7 | 5085.9 | 5167.7 | 4943.0 | 5311.8 | 5017.4 | 5015.8 | 5177.3 | 5098.4 | 114.32 | 2.24 |
|  | 50 | 2199.9 | 2218.4 | 2144.6 | 2258.0 | 2179.0 | 2160.5 | 2128.7 | 2201.2 | 2147.0 | 2117.6 | 2175.5 | 44.04 | 2.02 |
|  | 25 | 925.3 | 942.8 | 929.7 | 934.1 | 928.6 | 924.2 | 953.8 | 920.9 | 922.0 | 922.0 | 930.4 | 10.60 | 1.14 |
| EpCAM mRNA | 100 | 306.5 | 319.9 | 314.8 | 293.2 | 291.1 | 287.0 | 287.0 | 291.1 | 277.7 | 314.8 | 298.3 | 14.49 | 4.86 |
|  | 50 | 132.9 | 123.7 | 122.7 | 112.6 | 125.8 | 118.7 | 127.8 | 123.7 | 112.6 | 119.7 | 122.0 | 6.37 | 5.22 |
|  | 25 | 61.2 | 56.2 | 62.2 | 62.2 | 62.2 | 60.2 | 65.2 | 56.2 | 59.2 | 57.2 | 60.2 | 3.00 | 4.98 |
| GPC3 mRNA | 100 | 268.5 | 269.5 | 275.7 | 276.7 | 239.8 | 255.1 | 273.6 | 249.0 | 266.4 | 262.3 | 263.7 | 12.19 | 4.62 |
|  | 50 | 105.5 | 112.6 | 107.6 | 91.4 | 109.6 | 105.5 | 113.6 | 104.5 | 101.5 | 100.5 | 105.2 | 6.48 | 6.15 |
|  | 25 | 54.1 | 52.1 | 55.1 | 57.2 | 56.2 | 53.1 | 55.1 | 57.2 | 58.2 | 53.1 | 55.1 | 2.01 | 3.65 |
| PD-L1 mRNA | 100 | 108.6 | 114.6 | 113.6 | 106.6 | 118.7 | 100.5 | 104.5 | 109.6 | 104.5 | 109.6 | 109.1 | 5.44 | 4.99 |
|  | 50 | 50.1 | 47.1 | 50.1 | 50.1 | 52.1 | 52.1 | 47.1 | 49.1 | 51.1 | 52.1 | 50.1 | 1.89 | 3.78 |
|  | 25 | 20.0 | 23.0 | 19.0 | 18.0 | 20.0 | 22.0 | 20.0 | 19.0 | 18.0 | 17.0 | 19.6 | 1.84 | 9.39 |

The reproducibility of MRT-dPCR was evaluated by calculating the percent coefficient of variation (%CV) for EpCAM protein, GPC3 protein, ASGPR protein, EpCAM mRNA, GPC3 mRNA, and PD-L1 mRNA.

**Supplementary Table 6. LOB for each marker based on MRT-dPCR using artificial samples devoid of CTCs**

|  | EpCAM protein | GPC3 protein | ASGPR protein | EpCAM mRNA | GPC3 mRNA | PD-L1 mRNA |
| --- | --- | --- | --- | --- | --- | --- |
| Test 1 | 95.2 | 80.2 | 123.4 | 2.0 | 3.0 | 0.0 |
| Test 2 | 90.2 | 69.1 | 115.3 | 2.0 | 1.0 | 0.0 |
| Test 3 | 99.2 | 99.2 | 188.9 | 0.0 | 5.0 | 1.0 |
| Test 4 | 109.3 | 92.2 | 115.3 | 9.0 | 6.0 | 0.0 |
| Test 5 | 105.3 | 82.2 | 127.4 | 4.0 | 6.0 | 2.0 |
| Test 6 | 107.3 | 93.2 | 196.9 | 3.0 | 7.0 | 0.0 |
| Test 7 | 102.3 | 95.2 | 142.5 | 5.0 | 9.0 | 0.0 |
| Test 8 | 97.2 | 101.3 | 160.6 | 7.0 | 9.0 | 7.0 |
| Test 9 | 101.3 | 88.2 | 132.4 | 0.0 | 0.0 | 0.0 |
| Test 10 | 92.2 | 87.2 | 145.5 | 3.0 | 2.0 | 0.0 |
| Test 11 | 98.2 | 91.2 | 145.5 | 0.0 | 2.0 | 0.0 |
| Test 12 | 105.3 | 93.2 | 121.4 | 0.0 | 0.0 | 0.0 |
| Mean | 100.2 | 89.4 | 142.9 | 2.9 | 4.2 | 0.8 |
| SD | 6.0 | 8.9 | 27.1 | 2.9 | 3.3 | 2.0 |
| LOB  (copies) | 108.3 | 100.2 | 192.9 | 8.0 | 9.0 | 4.5 |

LOB, limit of blank.

**Supplementary Table 7. LOD of MRT-dPCR assay based on MRX-CTC chip for protein and mRNA detection.**

| Dilution | Standard (mL^-1^) | Total number of tests | No. of positive results | | | | | |
| --- | --- | --- | --- | --- | --- | --- | --- | --- |
|  |  |  | EpCAM  protein | GPC3  protein | ASGPR  protein | EpCAM  mRNA | GPC3  mRNA | PD-L1  mRNA |
|  | 100 | 10 | 10 | 10 | 10 | 10 | 10 | 10 |
| 2× | 50 | 10 | 10 | 10 | 10 | 10 | 10 | 10 |
| 2× | 25 | 10 | 10 | 10 | 10 | 10 | 10 | 10 |
| 2× | 12.5 | 10 | 10 | 10 | 10 | 10 | 10 | 9 |
| 2× | 6.25 | 15 | 14 | 12 | 15 | 9 | 10 | 7 |
| 2× | 3.125 | 15 | 13 | 9 | 14 | 7 | 6 | 5 |
| LOD (mL^-1^) | | | 6.5 | 9.3 | 3.2 | 11.4 | 10.3 | 15.1 |

The positive results for each marker were determined based on the LOB for that marker. The LOD for each marker was calculated through Probit regression analysis based on the number of positive results. LOB, Limit of Blank; LOD, Limit of Detection.

**Supplementary Table 8. Summary of Detailed Results of ROC Curves for Different Markers Differentiating HD and HCC.**

| Marks | No. of total samples | AUC | Sensitivity | Specificity | Cut off value | 95% LCL | 95% UCL |
| --- | --- | --- | --- | --- | --- | --- | --- |
| EpCAM protein | 77 | 0.840 | 0.547 | 1.000 | 172.727 | 0.743 | 0.937 |
| GPC3 protein | 77 | 0.647 | 0.340 | 0.958 | 107.281 | 0.501 | 0.793 |
| ASGPR protein | 77 | 0.921 | 0.830 | 0.917 | 221.191 | 0.851 | 0.991 |
| EpCAM mRNA | 77 | 0.822 | 0.623 | 1.000 | 18.413 | 0.702 | 0.942 |
| GPC-3 mRNA | 77 | 0.844 | 0.736 | 0.875 | 9.767 | 0.741 | 0.946 |
| PDL1 mRNA | 77 | 0.539 | 0.415 | 0.792 | 5.522 | 0.392 | 0.685 |

The ROC curves for each marker are briefly summarized in **Supplementary Fig. 6**

**Supplementary Table 9. Performance metrics comparison of Support Vector Classification (SVC) with different kernel functions for classification tasks.**

| Method | Accuracy | Precision | Recall | F1 |
| --- | --- | --- | --- | --- |
| SVC with linear | 0.506 | 0.642 | 0.693 | 0.499 |
| SVC with poly | 0.727 | 0.779 | 0.741 | 0.72 |
| SVC with GSNB | 0.649 | 0.688 | 0.662 | 0.641 |
| SVC with rbf | 0.74 | 0.799 | 0.759 | 0.735 |

## Supplementary Notes

Multi-channel process-based analysis model (M-PAM) encompasses algorithms for fluorescence data extraction (**Supplementary Note 1**), noise reduction, analysis, and key parameter extraction (**Supplementary Note 2**). Support Vector Classification (SVC) algorithms with different kernel functions in machine learning are detailed in **Supplementary Note 3**. All code is executed in Python.

### Supplementary Note 1

Fluorescence data extraction algorithm：

import numpy as np

import cv2

import pandas as pd

import threading

import math

import glob

import json

import os

import struct

# Template for the chip

template_X = []

template_Y = []

begin_X1 = 10

begin_Y1 = 10

begin_X2 = 15

begin_Y2 = 42

interval_X1 = 24.39

interval_X2 = 24.39

interval_Y = 58.49

for i in range(92):

for j in range(32):

template_X.append(begin_X1 + int(i * interval_X1 - j * 0.06))

template_X.append(begin_X2 + int(i * interval_X2 - j * 0.06))

template_Y.append(begin_Y1 + int(j * interval_Y))

template_Y.append(begin_Y2 + int(j * interval_Y))

start_p = 0

end_p = 0

def draw_template(lt, lb, rt):

global template_X

global template_Y

x_step = (lb[1] - lt[1]) / 159

y_step = (rt[0] - lt[0] - 16) / 63

angle = math.atan2((lb[0] - lt[0]), lb[1] - lt[1])

template_X = []

template_Y = []

x0 = lt[0]

y0 = lt[1]

x1 = x0 + 15

y1 = y0 + 7

for i in range(160):

for j in range(64):

if j < 40:

para = 1

else:

para = 1

template_X.append(x0 + int(j * y_step * math.cos(angle) + i * math.sin(angle) * x_step + para * (0 - abs(i * j)) * 0.0008))

template_X.append(x1 + int(j * y_step * math.cos(angle) + i * math.sin(angle) * x_step + para * (0 - abs(i * j)) * 0.0008))

template_Y.append(

y0 + int(i * x_step * math.cos(angle) - j * math.sin(angle) * y_step + (80 - abs(i - 80)) * 0.04 + max(j, 0) * 0.05))

template_Y.append(

y1 + int(i * x_step * math.cos(angle) - j * math.sin(angle) * y_step + (80 - abs(i - 80)) * 0.04 + max(j, 0) * 0.05))

return template_X, template_Y

# Extract points inside the circle

def get_point_in_circle(img, center_x, center_y):

points = []

for i in range(-3, 4):

for j in range(-3, 4):

if i * i + j * j <= 25:

if center_y - j > 0 and center_x - i > 0 and center_y + j < img.shape[0] and center_x + i < img.shape[1]:

points.append(img[center_y + j, center_x + i])

return points

# Sort contours

def sort_contours(contours):

c_moments = [[cv2.moments(c)['m00'], cv2.moments(c)['m01'], cv2.moments(c)['m10']] for c in contours]

position = [[int(m[2] / m[0]), int(m[1] / m[0])] for m in c_moments]

a = sorted(zip(contours, position), key=lambda x: x[1][0] + x[1][1]) # Top left

b = sorted(zip(contours, position), key=lambda x: -x[1][1] + x[1][0]) # Bottom left

c = sorted(zip(contours, position), key=lambda x: x[1][1] - x[1][0]) # Bottom right

return a, b, c

def find_chip(img):

global start_p, end_p

X = []

Y = []

def get_mouse_position(event, x, y, flags, param):

if event == cv2.EVENT_LBUTTONDBLCLK:

if len(X) < 3:

print(x, y)

X.append(x)

Y.append(y)

cv2.namedWindow('Image', cv2.WINDOW_NORMAL | cv2.WINDOW_KEEPRATIO)

cv2.setMouseCallback('Image', get_mouse_position)

cv2.imshow('Image', img)

cv2.waitKey(0)

start_p = [Y[0], X[0]]

end_p = [Y[1], X[1]]

# Image processing with filtering

def image_adaptive(img_real):

# Sharpen

clahe = cv2.createCLAHE(clipLimit=1.0, tileGridSize=(35, 35))

res = clahe.apply(img_real)

kernel = np.array([[0, -1, 0], [-1, 5, -1], [0, -1, 0]], np.float32)

dst = cv2.filter2D(res, -1, kernel=kernel)

dst[dst > 254] = 0

# Gaussian filtering

img_filter = cv2.GaussianBlur(dst, (3, 3), 0)

# Adaptive thresholding

img_adaptive = cv2.adaptiveThreshold(img_filter, 45535, cv2.ADAPTIVE_THRESH_GAUSSIAN_C, cv2.THRESH_BINARY, 93, -12)

# Opening operation

kernel = cv2.getStructuringElement(cv2.MORPH_RECT, (25, 25))

img_open = cv2.morphologyEx(img_adaptive, cv2.MORPH_CLOSE, kernel)

# Connected component filling

img_seedFilling = img_open.copy()

img_seedFilling = cv2.threshold(img_seedFilling, 15, 255, cv2.THRESH_BINARY)[1]

# Remove noise

img_adaptive1 = cv2.bitwise_and(img_adaptive, img_open)

return img_adaptive1

# Hole positioning

def find_TopAndAngle(image, auto):

global rox_L_x

global rox_L_y

global rox_R_x

global rox_R_y

global angle

global start_row, start_col, end_row, end_col

if start_p == 0:

img_real = image[0:2400, 000:-300]

else:

img_real = image[start_p[0]:end_p[0], start_p[1]:end_p[1]]

# Automatic corner detection

if auto == 1:

img_adaptive1 = image_adaptive(img_real)

contours, cnt = cv2.findContours(img_adaptive1.copy(), cv2.RETR_EXTERNAL,

cv2.CHAIN_APPROX_SIMPLE)

contour_useable = []

for item in contours:

if cv2.contourArea(item) > 25 and cv2.contourArea(item) < 85:

contour_useable.append(item)

a, b, c = sort_contours(contour_useable)

img = cv2.drawContours(img_real, contour_useable, -1, 255, 1)

cv2.imwrite('./images/' + str(1) + '.tiff', img)

try:

left_top = a[0][1]

left_bottom = b[0][1]

right_top = c[0][1]

except:

left_top = [15, 15]

left_bottom = [15, 15]

right_top = [15, 15]

# Manual corner detection

elif auto == 0:

X = []

Y = []

def get_mouse_position(event, x, y, flags, param):

if event == cv2.EVENT_LBUTTONDBLCLK:

if len(X) < 3:

print(x, y)

X.append(x)

Y.append(y)

cv2.namedWindow('Corner Point', cv2.WINDOW_NORMAL | cv2.WINDOW_KEEPRATIO)

cv2.setMouseCallback('Corner Point', get_mouse_position)

cv2.imshow('Corner Point', img_real[:300, :300])

cv2.waitKey(0)

left_top = [X[0], Y[0]]

cv2.namedWindow('Corner Point', cv2.WINDOW_NORMAL | cv2.WINDOW_KEEPRATIO)

cv2.setMouseCallback('Corner Point', get_mouse_position)

cv2.imshow('Corner Point', img_real[1800:, :300])

cv2.waitKey(0)

left_bottom = [X[1], Y[1] + 1800]

cv2.namedWindow('Corner Point', cv2.WINDOW_NORMAL | cv2.WINDOW_KEEPRATIO)

cv2.setMouseCallback('Corner Point', get_mouse_position)

cv2.imshow('Corner Point', img_real[:300, 1600:])

cv2.waitKey(0)

right_top = [X[2] + 1600, Y[2]]

# Draw positioning points

print(left_top, right_top, left_bottom)

img = cv2.circle(img_real, (left_top[0], left_top[1]), 10, 255, 2)

img = cv2.circle(img_real, (left_bottom[0], left_bottom[1]), 10, 255, 2)

img = cv2.circle(img_real, (right_top[0], right_top[1]), 10, 255, 2)

cv2.imwrite('./images/1_' + str(0) + '.tiff', img)

template_X, template_Y = draw_template(left_top, left_bottom, right_top)

return template_X, template_Y

def label_chip_image(data, img):

# Read the header and move the pointer

X = template_X.copy()

Y = template_Y.copy()

num_channels = data[0][4] # This is the number of channels

y_pixel_length = int((len(data[0]) - 6) / 4 / num_channels) # This is the number of points on the y-axis

x_pixel_length = len(data) # This is the number of points on the x-axis

t_min = 0

threshold_num = 1

global start_p, end_p

for i in range(y_pixel_length):

for j in range(x_pixel_length):

for k in range(num_channels):

# Signal intensities for each point

value = data[j][(6 + k * y_pixel_length * 4 + 4 * i + 2):(6 + k * y_pixel_length * 4 + 4 * i + 4)]

value = struct.unpack('>h', value)[0] / 10000

t_min = t_min + value

if t_min < 0.3:

cv2.circle(img, (X[i], Y[i]), 2, 0, 3)

elif t_min < 0.7:

cv2.circle(img, (X[i], Y[i]), 3, 0, 3)

else:

cv2.circle(img, (X[i], Y[i]), 4, 0, 3)

t_min = 0

cv2.imwrite('./images/result/' + str(0) + '_final.tiff', img)

def parse_chip_file(file_name, file_img):

file_name, file_img = file_name, file_img

with open(file_name, 'rb') as f:

content = f.readlines()

chip_info = []

for line in content:

chip_info.append(line)

label_chip_image(chip_info, file_img)

if __name__ == '__main__':

# Read all tiff files

files = glob.glob('./images/*.tiff')

files_img = []

for f in files:

file_img = cv2.imread(f, 0)

files_img.append(file_img)

# Read all binary files

files_data = glob.glob('./data/*/*.dat')

# Match one-to-one and start processing

for i in range(len(files_data)):

print('Start processing the file:', files_data[i])

template_X, template_Y = find_TopAndAngle(files_img[i], 1)

parse_chip_file(files_data[i], files_img[i])

print('The file has been successfully processed:', files_data[i])

### Supplementary Note 2

Algorithm for data denoising, analysis, and key parameter extraction：

import pandas as pd

import numpy as np

import glob

from scipy import signal, interpolate

from scipy.optimize import curve_fit, differential_evolution

from PyEMD import EMD, Visualisation

def sigmoid(x, a, b, c, d):

return a / (1 + np.exp(-b * (x - c))) + d

def sumOfSquaredError(parameterTuple, *data):

x, y = data

val = sigmoid(x, *parameterTuple)

return np.sum((y - val) ** 2.0)

def generate_Initial_Parameters(xData, yData): # Generate initial fitting parameters

# min and max used for bounds

args = (xData, yData)

maxX = max(xData)

minX = min(xData)

maxY = max(yData)

minY = min(yData)

parameterBounds = []

parameterBounds.append([minY, maxY]) # search bounds for a

parameterBounds.append([0, 1]) # search bounds for b

parameterBounds.append([minX, maxX]) # search bounds for c

parameterBounds.append([minY, maxY]) # search bounds for d

# "seed" the numpy random number generator for repeatable results

result = differential_evolution(sumOfSquaredError, parameterBounds, args=args, seed=3)

return result.x

def find_valley(data): # Find valley

min_index = signal.argrelmin(data)

for index in min_index[0]:

if len(data) > index > 6:

return int(index) - 4

return 10

def analyse_pcr(sub_subpath):

file_name = sub_subpath + './data.csv'

try:

df_ori = pd.read_csv(file_name)

df_ori = df_ori.dropna()

except Exception as ex:

return ex

df_r2 = df_ori[df_ori['round'] == 2] # Use the second round for analysis

r = df_r2['value'].rolling(window=20, center=True)

mp1 = r.mean() + 3. * r.std() # Upper limit: 3 times standard deviation

mp1 = mp1.fillna(60000)

mp2 = r.mean() - 3. * r.std()

mp2 = mp2.fillna(0)

df_invalid_index = df_r2[(df_r2['value'] > mp1) | (df_r2['value'] < mp2)].index # Outlier labels

invalid_hole = df_r2.loc[df_invalid_index, 'hole'].tolist()

if sub_subpath[-1] == '1':

threshold_rate = 2.3

else:

threshold_rate = 2.5

df_res = pd.DataFrame()

# def function3(x, a, b, c):

# return a * x*x + b*x + c

def function3(x, a, b):

return 1.78709 * np.power(x, 2) * a - 206.7096 * np.power(x, 1) * a + 9000 + b

for key, value in df_ori.groupby('hole'):

# Initial judgment

if key == '45-62':

print(key)

if len(value) < 5:

continue

df_res_tmp = {'hole': [key]}

value = value.sort_values(by='round')

value = value.reset_index(drop=True)

value = value.fillna(0)

# Baseline correction

baseline = np.nanmean(value['value'][10:15])

a, b = signal.butter(3, 0.2, 'low')

sg = signal.savgol_filter(value['value'], 50, 3, mode='nearest')

baseline = int(np.nanmean(sg[10:15]))

endline = np.nanmean(sg[-3:])

slope = (np.nanmean(sg[-3:]) - np.nanmean(sg[:3])) / len(sg)

if baseline == 0:

add_rate = 0

else:

add_rate = (max(sg[-3:]) - baseline) / baseline

add_delta = (max(sg[-3:]) - baseline)

df_res_tmp['add rate'] = add_rate

df_res_tmp['adddelta'] = add_delta

df_res_tmp['slope'] = slope

df_res_tmp['diff'] = 0

df_res_tmp['CV'] = np.std(value['value']) / np.mean(value['value'])

value['filt_value'] = sg

df_res_tmp['ori_point'] = [[int(x) for x in value['value']]]

df_res_tmp['eff'] = 0

if add_rate < threshold_rate:

df_res_tmp['type'] = 0

df_res_tmp['baseline'] = baseline

df_res_tmp['CT'] = 'negative'

df_res_tmp['point'] = [sg]

else:

emd = EMD()

length = len(value)

df_res_tmp['type'] = 1

try:

emd.emd(np.array(value['filt_value']))

imfs, res = emd.get_imfs_and_residue()

valley = find_valley(imfs[-1])

except:

high = value.loc[length - 1, 'filt_value'] - value.loc[0, 'filt_value']

increaseLine = pd.Series(np.arange(0, length))

increaseLine = increaseLine * high / length

imfs = value['value'] - increaseLine

valley = find_valley(np.array(imfs))

df_res_tmp['baseline'] = baseline

value.loc[:valley, 'filt_value'] = value.loc[valley, 'filt_value']

xData = np.arange(0, length).tolist()

yData = (value['filt_value']).tolist()

parm1 = [max(yData), 1, max(xData), min(yData)]

try:

fittedParameters, pcov = curve_fit(sigmoid, xData, yData, parm1, maxfev=1500)

sg = sigmoid(xData, *fittedParameters)

sg = [round(x, 4) for x in sg]

df_res_tmp['point'] = [sg]

xData = np.arange(0, length, 0.1)

interpolated = sigmoid(xData, *fittedParameters)

interpolated = pd.Series(interpolated)

data_cha = interpolated - interpolated.shift(1)

data_cha2 = data_cha - data_cha.shift(1)

ct = round(np.argmax(data_cha2) / 10, 4)

bs_line = sg[15]

df_res_tmp['eff'] = (sg[int(ct + 1)] - sg[int(ct)])

if ct > 60:

if np.mean(sg[30:35]) < 20:

df_res_tmp['CT'] = 'negative'

df_res_tmp['type'] = 0

else:

df_res_tmp['CT'] = ct

elif ct < 0:

df_res_tmp['CT'] = 'negative'

df_res_tmp['type'] = 0

else:

df_res_tmp['CT'] = ct

except:

df_res_tmp['point'] = [sg]

df_res_tmp['type'] = -1

print('Fitting failed')

if key in invalid_hole:

df_res_tmp['type'] = -1

df_res_tmp = pd.DataFrame(df_res_tmp)

df_res = pd.concat([df_res, df_res_tmp], ignore_index=True)

print(file_name.split('.csv')[0], ' analyze finished')

filepath = file_name.split('data.csv')[0] + 'statistics.xlsx'

writer = pd.ExcelWriter(filepath, engine='openpyxl')

df_threshold = {'threshold': [threshold_rate]}

df_threshold = pd.DataFrame(df_threshold)

df_res.to_excel(writer, 'sheet1')

df_threshold.to_excel(writer, 'threshold', index=None)

writer.save()

writer.close()

def analyse_melt(sub_subpath):

return True

def data_analyse(path, exp_num):

paths = glob.glob(path + '/*')

for subpath in paths:

subpaths = glob.glob(subpath + '/*')

for sub_subpath in subpaths:

analyse_pcr(sub_subpath)

# # Categorical analysis

# if sub_subpath.split('_')[-1] == 'pcr':

# analyse_pcr(sub_subpath)

# elif sub_subpath.split('_')[-1] == 'melt':

# analyse_melt(sub_subpath)

return True

if __name__ == '__main__':

data_analyse(r'F:\DEBUG PHOTO\rdPCR_v1\2024_07_11_17_26_23', '1234')

### Supplementary Note 3

Support vector classification (SVC) algorithm:

import numpy as np

import pandas as pd

from sklearn import svm

from sklearn.naive_bayes import GaussianNB

from matplotlib import pyplot as plt

from sklearn.metrics import accuracy_score, precision_score, recall_score, f1_score

def make_meshgrid(x, y, h=.02):

"""Create a mesh grid to plot.

Parameters

----------

x: Data to create grid for x-axis

y: Data to create grid for y-axis

h: Optional step size for the grid

Returns

-------

xx, yy : ndarrays

"""

x_min, x_max = x.min() - 0.1, x.max() + 0.1

y_min, y_max = y.min() - 0.1, y.max() + 0.1

xx, yy = np.meshgrid(np.arange(x_min, x_max, h),

np.arange(y_min, y_max, h))

return xx, yy

def plot_contours(clf, xx, yy, **params):

"""Plot the decision boundaries of a classifier.

Parameters

----------

ax: Matplotlib subplot object

clf: A classifier

xx: Meshgrid ndarray for x-axis

yy: Meshgrid ndarray for y-axis

params: Dictionary of parameters to pass to contourf, optional

"""

Z = clf.predict(np.c_[xx.ravel(), yy.ravel()])

Z = Z.reshape(xx.shape)

out = plt.contourf(xx, yy, Z, **params)

return out

# dataframe = pd.read_excel(r'C:\Users\18561\Documents\WeChat Files\yangqi699818\FileStorage\File\2024-08\traindata.xlsx')

# X = dataframe[['feature3', 'feature4']]

# Y = dataframe['label']

# X = np.array(X)

#

# sample_weight_last_ten = np.ones(len(X))

# # Assign higher weights to outliers

# # sample_weight_last_ten[3:4] *= 3

# # sample_weight_last_ten[12] *= 3

# sample_weight_last_ten[:24] *= 5

# # Create an SVM instance and fit the data

# clf = svm.SVC(kernel='poly') #svm.NuSVC(gamma='auto')

#

# # clf = GaussianNB()

# clf.fit(X, Y, sample_weight=sample_weight_last_ten)

#

# predict = clf.predict(X)

# Y = np.array(Y)

# # Accuracy

# accuracy = accuracy_score(predict, Y)

# print("Accuracy:", accuracy)

# # Precision

# precision = precision_score(predict, Y, average='macro')

# print("Precision:", precision)

# # Recall

# recall = recall_score(predict, Y, average='macro')

# print("Recall:", recall)

# # F1 Score

# f1 = f1_score(predict, Y, average='macro')

# print("F1 Score:", f1)

#

# # Plot the decision boundary. For this, we will assign a color to each

# # point in the mesh [x_min, x_max] x [y_min, y_max].

#

# X0, X1 = X[:, 0], X[:, 1]

# xx, yy = make_meshgrid(X0, X1)

# plot_contours(clf, xx, yy,

# cmap=plt.cm.coolwarm, alpha=0.8)

#

# # Plot training points

# plt.scatter(X[:, 0], X[:, 1], c=Y, cmap=plt.cm.Paired, edgecolors='k')

# plt.title('SVC with GSNB kernel')

# plt.axis('tight')

# plt.xlabel('EpCAM MRNA/ASGPR protein')

# plt.ylabel('EpCAM protein/ASGPR protein')

# # plt.xlabel('GPC-3 mRNA/ASGPR protein')

# # plt.ylabel('GPC-3 protein/ASGPR protein')

# plt.show()

dateframe = pd.read_excel(r'C:\work\image tools\Data\para2.xlsx')

columns = dateframe.columns

index = dateframe.iloc[:,0]

X = []

Y = []

for x in range(1,len(columns)):

for y in range(1,len(index)):

if dateframe.iloc[y,x] - dateframe.iloc[y-1,x]==-1:

X.append(float(columns[x]))

Y.append(index[y])

df1 = {'X': X, 'Y': Y}

df1 = pd.DataFrame(df1)

df1.to_excel('curve.xlsx')

## Reference

1 Gao, X. *et al.* High filling rate digital PCR through-hole array chip with double independent S-shaped flow channels. *Biomicrofluidics* **14**, 034109 (2020).
